# *In Vivo* Sarcomere Lengths Become More Non-uniform upon Activation in Intact Whole Muscle

**DOI:** 10.3389/fphys.2017.01015

**Published:** 2017-12-07

**Authors:** Eng Kuan Moo, Timothy R. Leonard, Walter Herzog

**Affiliations:** Human Performance Laboratory, Faculty of Kinesiology, University of Calgary, Calgary, AB, Canada

**Keywords:** second harmonic generation microscopy, active contraction, *in vivo*, non-uniformity, force-length relationship, skeletal muscle properties, sarcomere instability

## Abstract

The sarcomere force-length relationship has been extensively used to predict muscle force potential. The common practice is to measure the mean sarcomere length (SL) in a relaxed muscle at a single location and at a given length, and this mean SL is assumed to represent the SLs at other locations across the muscle. However, in a previous study, we found that SLs are highly non-uniform across an intact passive muscle. Moreover, SL non-uniformity increases during activation in single myofibril experiments. Myofibrils lack some structural proteins that comprise an intact muscle, and therefore, the increased SL dispersion upon activation seen in myofibrils may not occur in intact whole muscle. The objectives of the current study were (i) to measure the distribution of SLs in an activated intact muscle; and (ii) to assess the feasibility of using the mean SL measured at a specific location of the muscle to predict muscle force. Using state-of-the-art multi-photon microscopy and a miniature tendon force transducer, *in vivo* sarcomeres in the mouse tibialis anterior were imaged simultaneously with muscle force during isometric tetanic contractions. We found that *in vivo* SL dispersion increased substantially during activation and reached average differences of ~1.0 μm. These differences in SL are associated with theoretical force differences of 70–100% of the maximal isometric force. Furthermore, SLs measured at a single location in the passive muscle were poor predictors of active force potential. Although mean SLs in the activated muscle were better predictors of force potential, predicted forces still differed by as much as 35% from the experimentally measured maximal isometric forces.

## Introduction

Sarcomeres are subcellular organelles that act as the basic force producing units in muscles. A sarcomere is composed of the contractile filaments myosin (anisotropic band, A-band) and actin (isotropic band, I-band) as well as a series of structural proteins including titin, nebulin, and desmin. The arrays of serially-connected sarcomeres give skeletal muscle its characteristic striated appearance. The micro-scale striation patterns are good indicators of myofibril integrity and are useful in diagnosing muscle pathologies (Plotnikov et al., 2008; Ralston et al., 2008). More importantly, the striation periodicity, or the corresponding instantaneous sarcomere length (SL) and the rate of change in SL, have been identified as important indicators of the functional properties (e.g., force, power) of a muscle (Hill, 1938; Gordon et al., 1966; Lutz and Rome, 1994; Burkholder and Lieber, 1998).

SL and SL non-uniformities have been crucial for understanding and explaining basic muscle properties (Hill, 1953; Huxley and Peachey, 1961; Morgan, 1994) and muscle function within the constraints of animal bodies (Cutts, 1988; Mai and Lieber, 1990; Lutz and Rome, 1994). According to the classic cross-bridge theory, the maximal steady-state isometric force produced by a muscle depends on the overlap between thick and thin myofilaments, which depends on the SL (Gordon et al., 1966). The theoretical force-length (FL) curve can be derived from the lengths of the actin and myosin filaments (Gordon et al., 1966; Herzog et al., 1992) and has been validated in single muscle fiber (Gordon et al., 1966; ter Keurs et al., 1978; Edman, 1999) and single myofibril (Bartoo et al., 1993; Leonard and Herzog, 2010; Rassier and Pavlov, 2010) experiments. However, the relationship between force and the corresponding SLs has never been validated for whole muscles due to the technical difficulties in visualizing individual SLs in activated muscle. Nevertheless, the mean sarcomere length has been used frequently to infer the amount of force and power a muscle can generate (Rack and Westbury, 1969; Cutts, 1988; Mai and Lieber, 1990; Lutz and Rome, 1994; Vaz et al., 2012). Though convenient, the main caveat of such practice is that it neglects the great non-uniformity of SLs (also referred to as dispersion of SLs) in neighboring sarcomeres at a given location within a muscle, and the differing SLs and SL changes at different locations of a muscle (Llewellyn et al., 2008; Moo et al., 2016).

Typically, SLs for a given muscle are measured at a single spot, often in the mid-belly of the muscle, and at a given muscle length (Rack and Westbury, 1969; Cutts, 1988; Mai and Lieber, 1990; Lutz and Rome, 1994; Vaz et al., 2012). It is then assumed implicitly that the SLs measured at this location represent the SLs at other locations within the muscle, and the FL, force-velocity and power-velocity properties of muscles are implied based on these single SL measurements (Rack and Westbury, 1969; Cutts, 1988; Lutz and Rome, 1994; Burkholder and Lieber, 2001; Vaz et al., 2012). However, it has been shown throughout the muscle hierarchy, from single myofibrils (Leonard and Herzog, 2010; Johnston et al., 2016), to single fibers (Huxley and Peachey, 1961; Infantolino et al., 2010), to whole muscles (Llewellyn et al., 2008; Cromie et al., 2013; Chen et al., 2016; Moo et al., 2016) that SLs vary dramatically within a muscle with the range of SLs easily covering 1.0 μm, which is equivalent to a range of 40% of the optimal SLs for average mammalian skeletal muscles (Huxley and Peachey, 1961; Walker and Schrodt, 1974; Herzog et al., 1992). Furthermore, SL dispersion has been shown to increase drastically in single myofibrils during activation and force production (Telley et al., 2006; Leonard and Herzog, 2010; Rassier and Pavlov, 2010; Johnston et al., 2016). Nevertheless, single myofibrils lack many of the structural proteins that provide stability to entire muscles. Thus, the increased SL non-uniformities during activation observed in myofibrils might not occur in whole muscles.

We used state-of-the-art second harmonic generation (SHG) imaging techniques to visualize sarcomeres in whole muscle (Llewellyn et al., 2008; Moo et al., 2016) and a miniaturized buckle type tendon force transducer (Walmsley et al., 1978; Hodgson, 1983; Abraham et al., 1985; Herzog and Leonard, 1991; Kaya et al., 2003, 2008) to directly measure the force generated in the mouse tibialis anterior muscle. Using these approaches, the purposes of this study were (i) to measure the distribution of SLs in an activated intact muscle and to compare the SL non-uniformities to those found in passive muscle; and (ii) to assess the feasibility of using the mean SL measured at a specific location in the muscle to predict maximal isometric muscle force. We hypothesized that SL non-uniformities are the same in the relaxed and activated muscle and that the mean SLs measured in the mid-belly of the tibialis anterior is a good predictor of the maximal isometric muscle force.

## Methods

### Animal preparation

All aspect of animal care and experimental procedures were carried out in accordance with the guidelines of the Canadian Council on Animal Care and were approved by the University of Calgary Life Sciences Animal Research and Ethics Committee. 10–12 week-old male C57BL6 mice (*n* = 9) were anesthetized using a 1–2% isoflurane/oxygen mixture. A cuff-type bipolar electrode of ~0.8 mm-diameter was implanted around the sciatic nerve to allow for controlled stimulation of the TA muscle in the anesthetized animals. The left proximal femur was fixed by a custom-made clamp at a knee flexion angle of ~120° (full knee extension = 180°), while the left foot was pinned to a movable base to allow for adjustment of tibialis anterior (TA) muscle tendon unit (MTU) length by changing the ankle joint angle. The skin over the left TA was opened and stretched to form a pool to accommodate a phosphate buffered saline solution that kept the muscle hydrated and allowed for imaging using water-immersion, multi-photon microscopy. The fascia over the TA was removed. All distal tendons around the ankle, except for the tendon of the TA were severed to eliminate the influence of other muscle groups during force measurement and to allow for a full range of motion of the ankle. A custom-made E-shaped tendon force transducer (Moo et al., 2017; see Supplementary Material S1) was implanted onto the distal TA tendon to measure force. The core temperature of the mice was maintained at ~30°C. All animals went through the same experimental protocol of imaging and electrical stimulation, and therefore no randomization was necessary.

### *In vivo* imaging of muscle under relaxed and activated conditions

**Figure 1:**
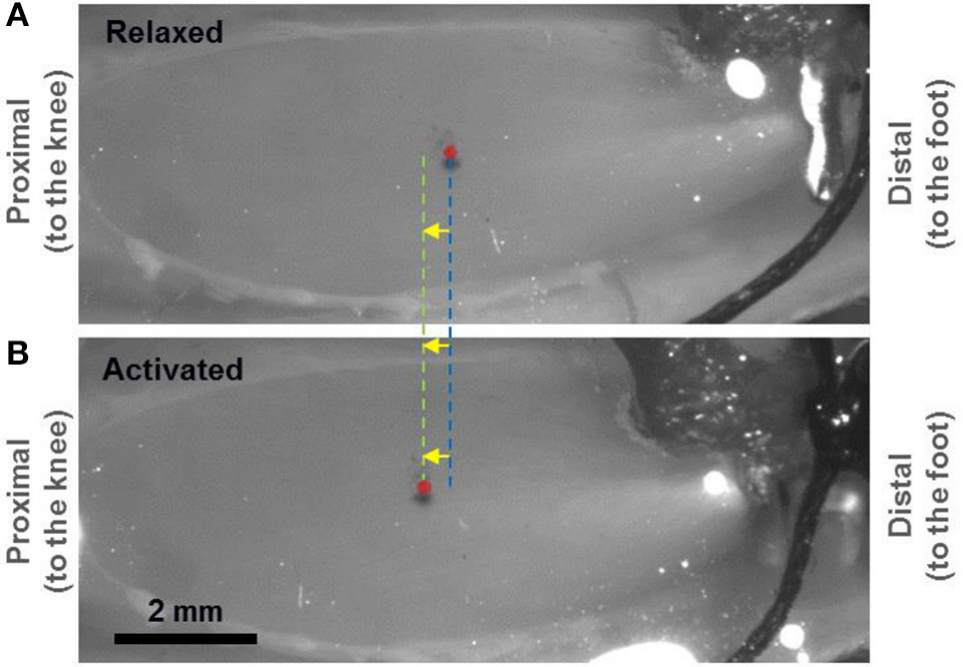
Light micrograph showing a mouse tibialis anterior (TA) muscle prepared for second harmonic generation (SHG) imaging of *in vivo* sarcomeres. A small portion of the implanted tendon force transducer can be seen at the distal end. The mid-belly of the TA, marked by the red circle, displaced ~375 μm proximally when going from the relaxed **(A)** to the activated **(B)** state. Two fluorescent markers that were separated by 1 mm were applied at the mid-belly of the TA (in proximity to the red circle) and observed under fluorescent light to measure the local displacement of the muscle caused by activation.

The challenge of this study was to image sarcomeres from a similar region in the relaxed and activated muscle, as the TA moved proximally during (isometric) activation (Figure 1). The TA was activated with a 600 ms continuous supra-maximal electrical stimulation (3 × α-motor neuron threshold, 60–70 Hz, 0.1 ms square wave pulse) of the sciatic nerve to produce fused tetanic contractions. In order to account for the proximal displacement of the TA during activation, two fluorescent markers separated by ~1 mm were attached on the mid-belly of the muscle using a 100 μm-diameter glass tip attached to a 3-axes linear micro-manipulator (Newport Corp., CA, USA). By observing the displacements of these two fluorescent markers through the eyepiece of a multi-photon excitation microscope (FVMPE-RS model, Olympus, Tokyo, Japan) under fluorescent light, measurements at the same location could be made for the passive and active muscle. A scanning area was selected between the two fluorescent markers and the TA muscle was imaged in its relaxed and active states. For details please be referred to the Supplementary Material S2.

SHG imaging of the TA was performed using an upright, multi-photon excitation microscope (FVMPE-RS model, Olympus, Tokyo, Japan) equipped with a wavelength-tunable (680–1,300 nm), ultrashort-pulsed (pulse width: <120 fs; repetition rate: 80 MHz) laser (InSight DeepSee-OL, Spectra-Physics, CA, USA) and a 25×/1.05 NA water immersion objective (XLPLN25XWMP2 model, Olympus, Tokyo, Japan). The TA was scanned using a laser wavelength of 800 nm. The resulting SHG signal emitted by the muscle was collected in the backward (epi-) direction using a band-pass filter at the harmonic frequency (FF01 400/40, Semrock Inc. NY, USA). The average power in the sample plane was set between 15 and 18 mW in order to acquire optimal images without damaging the muscle.

**Figure 2:**
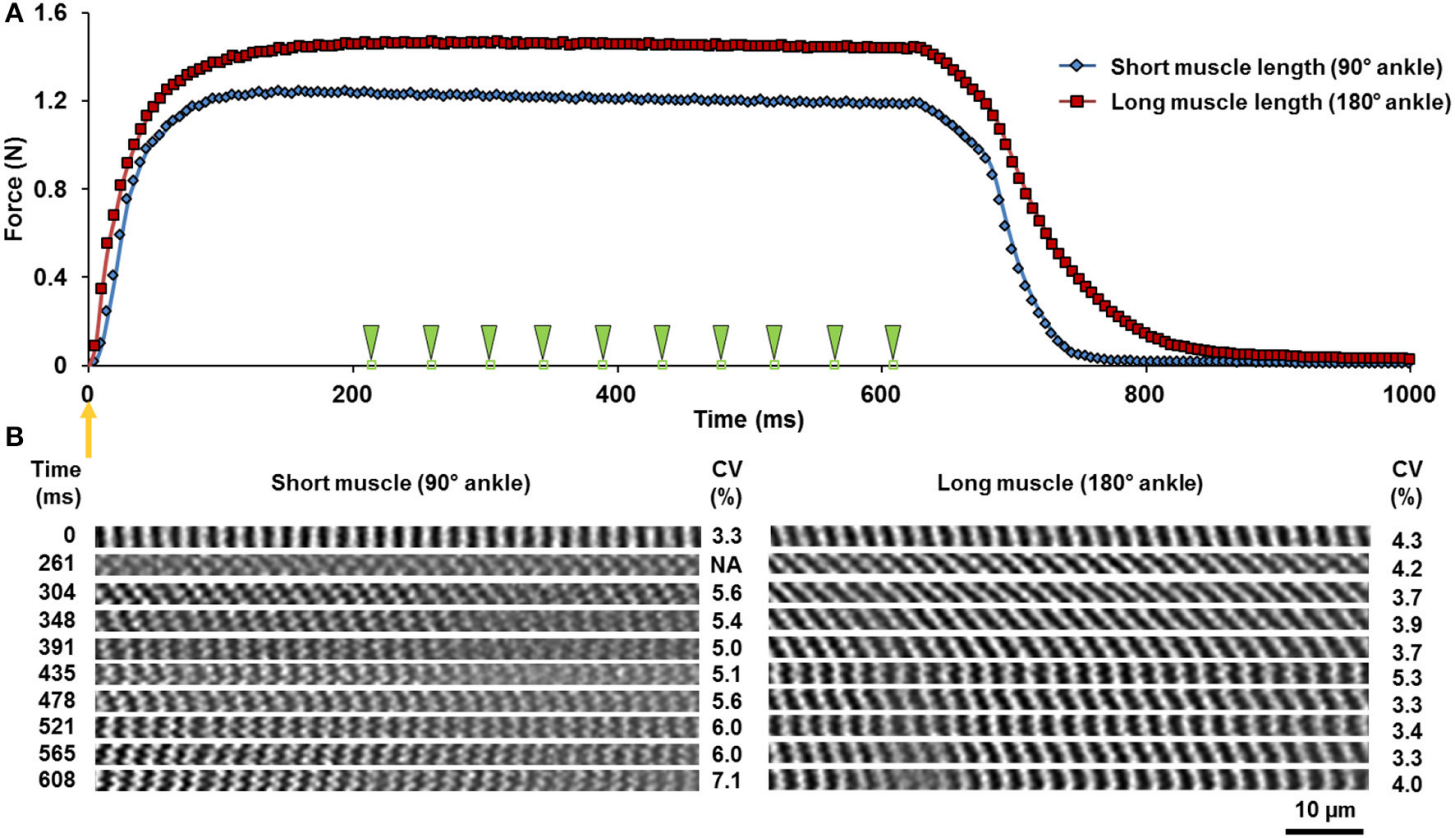
**(A)** Force-time curve during a 600 ms tetanic isometric contraction of the TA at the short (blue diamonds) and the long (red squares) lengths. The orange arrow indicates the time of muscle activation. The green arrowheads point to the times at which sarcomere images were acquired. **(B)** The corresponding time-series of the two-dimensional image bands of sarcomeres in the relaxed (0 ms) and the activated state. The A-bands appear as white bands in the images. The coefficients of variation (CV) of sarcomere lengths are shown for each image band. Note that the image band acquired at time 215 ms was excluded due to the image quality not meeting the selection criteria described in Methods *In Vivo* Imaging of Muscle under Relaxed and Activated Conditions.

Time-series, planar images were acquired in the horizontal plane (imaging area: 159 × 2.8 μm; pixel size: 0.2 μm; bit-depth: 12; dwell time: 2 μs) at a frame rate of 23 frames/s. Planar images of the TA were taken from the top 100 μm of the muscle. The SHG imaging of the muscle was performed at ankle angles of 90 and 180° flexion (full plantarflexion). These ankle angles will hereafter be denoted as short and long MTU length, respectively. Only images with good signal-to-noise ratio and minimal motion artifacts were included for image analysis. For the activated muscle, only images acquired during the steady-state phase of the tetanic contraction (between 200 and 600 ms of the contraction, Figure 2) were analyzed. No blinding was done as all images that fit the aforementioned inclusion criteria were processed similarly and objectively by image processing tools.

### Image analysis and sarcomere length measurement

Planar image bands of 14 pixels (2.8 μm) wide that contained 5–30 sarcomeres in series were selected. By assuming a myofibril diameter of ~1.3 μm, we could determine that each image band contained about two parallel-running myofibrils and therefore contained between 10 and 60 sarcomeres. Then, each image band was bandpass filtered using Fiji software (National Institutes of Health, MD, USA). The filtered image band was analyzed using custom-written MATLAB code that identified the centroids of the sarcomeric A-bands. Individual SLs were measured as the distance between adjacent A-band centroids (Cromie et al., 2013; Moo et al., 2016). Due to the spindle-like shape of the mouse TA, SLs were corrected for out of plane orientation using a through-thickness muscle image to ensure that SLs were measured along the epimysium (see Supplementary Material S3).

The imaging/stimulation trials were divided into four groups based on the muscle states (relaxed vs. activated) and the MTU lengths (short vs. long): (1) relaxed-short (43 trials); (2) activated-short (118 trials); (3) relaxed-long (41 trials); (4) activated-long (169 trials). For each trial in the activated muscle, 10 time-series images were acquired. The SLs measured from these time-series images were pooled, as there was no difference in SLs among them (see Results and **Figure 5**). The variables of interest including the mean, standard deviation (SD), coefficient of variation (CV = standard deviation/mean), shortest, longest, and length range (the difference between the longest and the shortest sarcomere in each image band) of SLs were derived from each image band. In total, the number of image bands analyzed in the relaxed-short, activated-short, relaxed-long, activated-long groups were 145, 505, 132, and 1,005 bands, respectively. We report the average values of these variables from all image bands acquired from the nine animals. In addition, we also grouped the data by contraction time and analyzed for changes of the mean values of the mean, CV, length range of SLs with contraction time. We also categorized the individual SLs as belonging to the ascending limb, plateau and descending limb of the theoretical sarcomere FL curve to study the activation-induced changes in length distribution on the FL curve. In each of the four groups, two weighted Gaussian curves were fitted to the resulting SL probability distribution function (PDF) using a Gaussian mixture model with variational inference (Blei and Jordan, 2006).

### Relationship between muscle force and sarcomere length

For every animal tested (*n* = 9), a Gaussian curve was fitted to the SL PDF using a Gaussian mixture model with variational inference (Blei and Jordan, 2006). The mean value of this fitted Gaussian curve was defined as representing the mean SL, which was then used to derive the relative muscle force (*F*_*r*_) from the theoretical FL curve (**Figure 6C**). The muscle force measured at 200 ms into activation (*F*_*m*_) was used for comparison with *F*_*r*_ as this force represents the isometric force at steady state with no apparent influence from muscle fatigue. The relationship between the measured (*F*_*m*_) and the theoretical force (*F*_*r*_) derived from the FL curve at the two muscle lengths is shown in Equation (1):

(1) $$\frac{F_{m\_long}}{F_{r\_long}}=\frac{F_{m\_short}}{F_{r\_short}}$$

The force generated by the TA at the short length was predicted using Equation (2) below and compared with the muscle force measured by the tendon transducer.

(2) $$F_{m\_short\_predicted}=F_{r\_short}\times \frac{F_{m\_long}}{F_{r\_long}}$$

The force prediction using the mean SL was defined as successful if the predicted force fell within ±5% of the measured force. Using Equation (2), the muscle force was also predicted for the pooled data from all nine animals. As there are two weighted Gaussian-fitted mean SLs for each muscle length, the resultant *F*_*r*_ was obtained using the same weights from the fitted Gaussian curves.

Likewise, *F*_*r*_ at the short muscle length can be predicted using Equation (3) below:

(3) $$F_{r\_short\_predicted}=F_{m\_short}\times \frac{F_{r\_long}}{F_{m\_long}}$$

Using the calculated *F*_*r*_ from Equation (3), the mean SL was predicted from the FL curve (**Figure 6C**) and compared with the mean SL measured by SHG imaging. For the cases in which the predicted *F*_*r*_ is not 1.0, and therefore the resulting force can occur at two SLs (i.e., on either side of the plateau region of the FL curve), the smallest difference between the predicted and the measured mean SL was reported. Also, for the case in which muscle force measured at the short length is higher than the force measured at the long length (forces in animal 1 and 2, see Supplementary Material S5), *F*_*m*_*long*_ and *F*_*r*_*long*_ were predicted in Equations (2) and (3) instead.

An error index, as defined in Equation (4), was also used to describe how well the mean SL predicts the experimentally measured force in the whole muscle.

(4) $$\text{Error index}=\frac{F_{m\_long}}{F_{r\_long}}-\frac{F_{m\_short}}{F_{r\_short}}$$

A calculation example using the pooled data from all nine animals is included in the Supplementary Material S4, for the interested readers.

### Statistical analysis

Statistical analyses were performed using SPSS (version 22, SPSS Inc. IL, USA). The means of the mean, local SD, local CV, longest, shortest, and length range of SLs were analyzed for condition effects using a generalized estimating equation (GEE, under Genlin procedures in SPSS) to take into account the correlated nature of the observations and the unbalanced study design. The relationships between contraction time and the mean SL, local CV, and length range of SLs were also individually analyzed with GEE. All statistical tests are two-sided with type I error, α, set at 0.05 level. Multiple comparisons were accounted for through Bonferroni adjusted *p*-values. As long as the data presented here are interval data, no assumption of normal distribution was needed for GEE statistical method. Also, as all the measurements were repeated measures, no estimate of variation within group was needed. Unless otherwise stated, results were expressed as estimated marginal means (EMM) ± standard error.

## Results

The average mass of the mice was 29 ± 4 g (mean ± SD). The total number of individual sarcomeres analyzed for each of the “relaxed-short,” “activated-short,” “relaxed-long,” and “activated-long” groups were 2,856 sarcomeres, 4,454 sarcomeres, 2,564 sarcomeres, and 8,270 sarcomeres, respectively.

**Figure 3:**
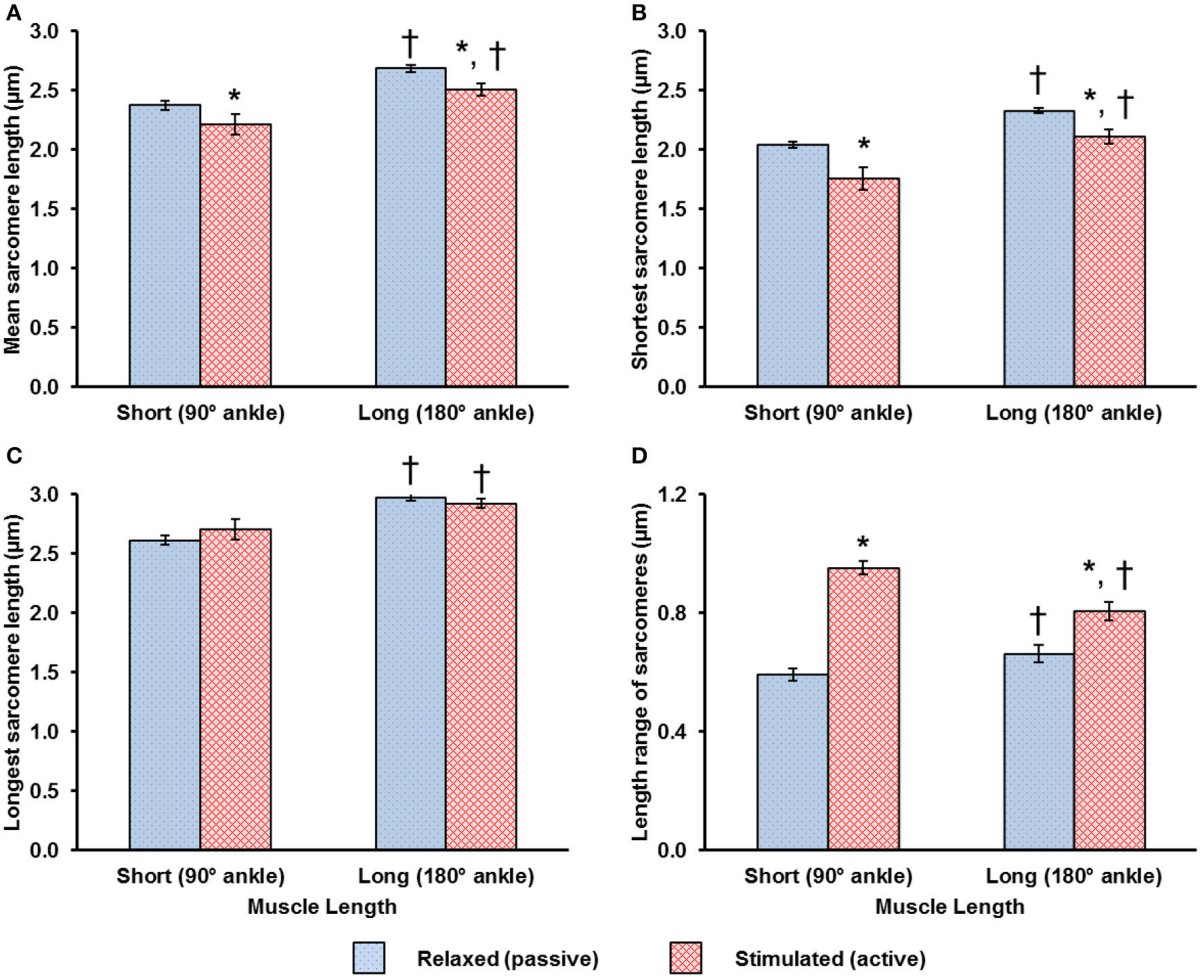
Mean values of **(A)** the mean sarcomere length (SL), **(B)** the shortest SL, **(C)** the longest SL, and **(D)** the SL range, which is defined as the difference between the longest and the shortest sarcomere in the individual image bands. These mean values were determined from 145, 505, 132, 1,005 image bands in the relaxed-short, activated short, relaxed-long, activated-long groups, respectively (see Methods). During activation, sarcomeres shortened by 7.1 and 6.7% in muscles at the short and long lengths, respectively **(A)**. Sarcomere shortening during activation resulted in a decrease in the shortest SL **(B)**, but did not influence the longest SL **(C)**. As a result, the SL range increased during activation. ^*^Indicates significant differences in mean SL, in shortest SL, in longest SL or in SL range compared with sarcomeres in the relaxed muscle at either the short or long length (*p* < 0.01). ^†^Indicates significant differences in mean SL, in shortest SL, in longest SL or in SL range compared with sarcomeres in either the relaxed or the activated muscle at the short length (*p* < 0.01).

When relaxed, sarcomeres in the short muscle were shorter (~2.4 μm) than sarcomeres in the long muscle (~2.7 μm; Figure 3A). During activation, sarcomeres shortened by 7.1 and 6.7% at the short and long muscle lengths, respectively (Figure 3A). The shortening in sarcomere lengths during activation resulted in a decrease in the shortest SL (Figure 3B), but did not influence the longest SL (Figure 3C). As a consequence, the SL range increased during activation (Figure 3D). In a region of 159 × 2.8 μm^2^, the local difference between the longest and shortest sarcomeres in activated muscle was as high as ~1 μm (Figure 3D).

**Figure 4:**
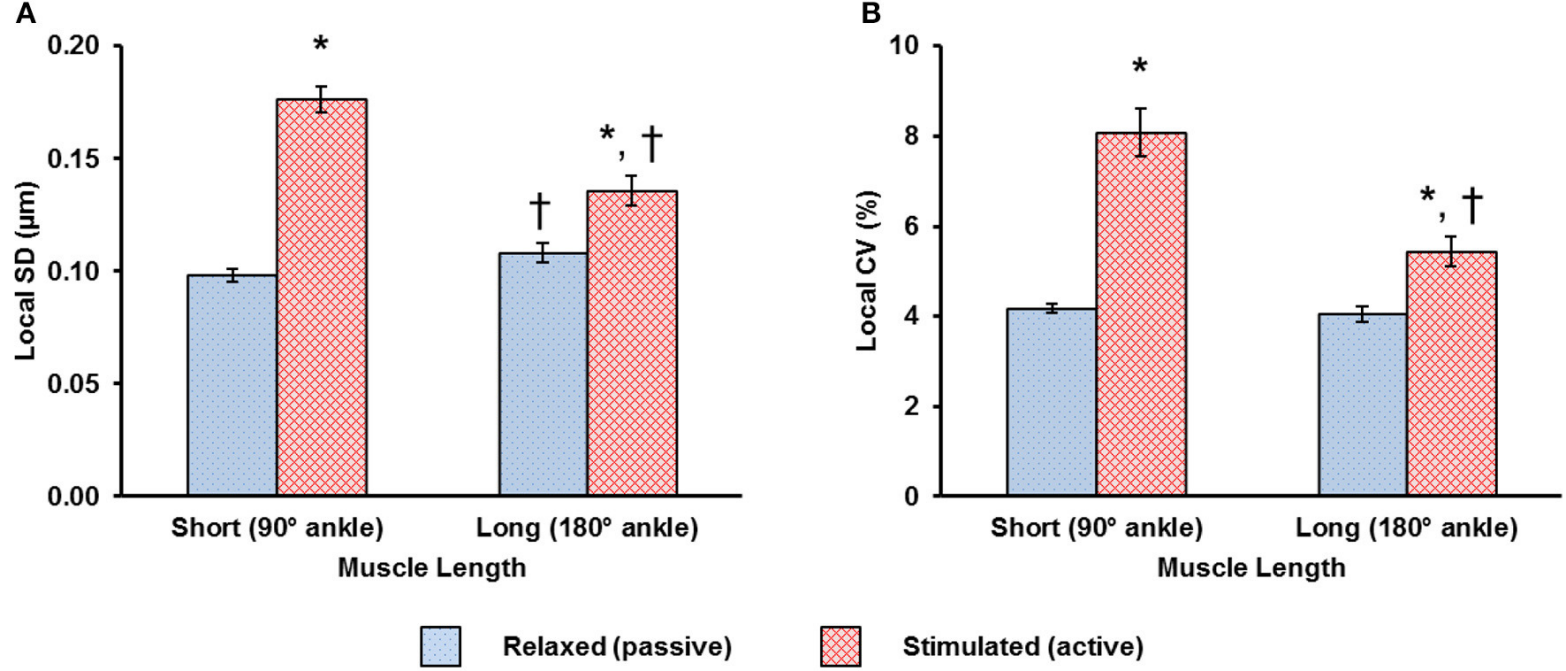
Changes in local dispersion of sarcomere lengths (SLs) due to activation are shown using **(A)** the local standard deviation (SD) and, **(B)** the local coefficient of variation (CV), and were measured at the short and long muscle lengths. Muscle activation resulted in increased dispersion in SLs. The increase in dispersion was more pronounced for sarcomeres at the short compared to the long muscle length. ^*^Indicates significant differences in local SD or in local CV compared with sarcomeres in the relaxed muscle at either the short or the long length (*p* < 0.01). ^†^Indicates significant differences in local SD or in local CV compared with sarcomeres in either the relaxed or the activated muscle at the short length (*p* < 0.01).

The local dispersion of the resting SLs was about 4% CV for the short and long muscle lengths (Figure 4). Activation resulted in an increased SL dispersion at the short and long muscle lengths (Figure 4). The SL dispersion increased by ~93% in the activated-short muscle compared to that observed in the relaxed-short muscle. At the long muscle length, however, this increase in SL dispersion from the relaxed to the activated state was only ~34% (Figure 4B).

**Figure 5:**
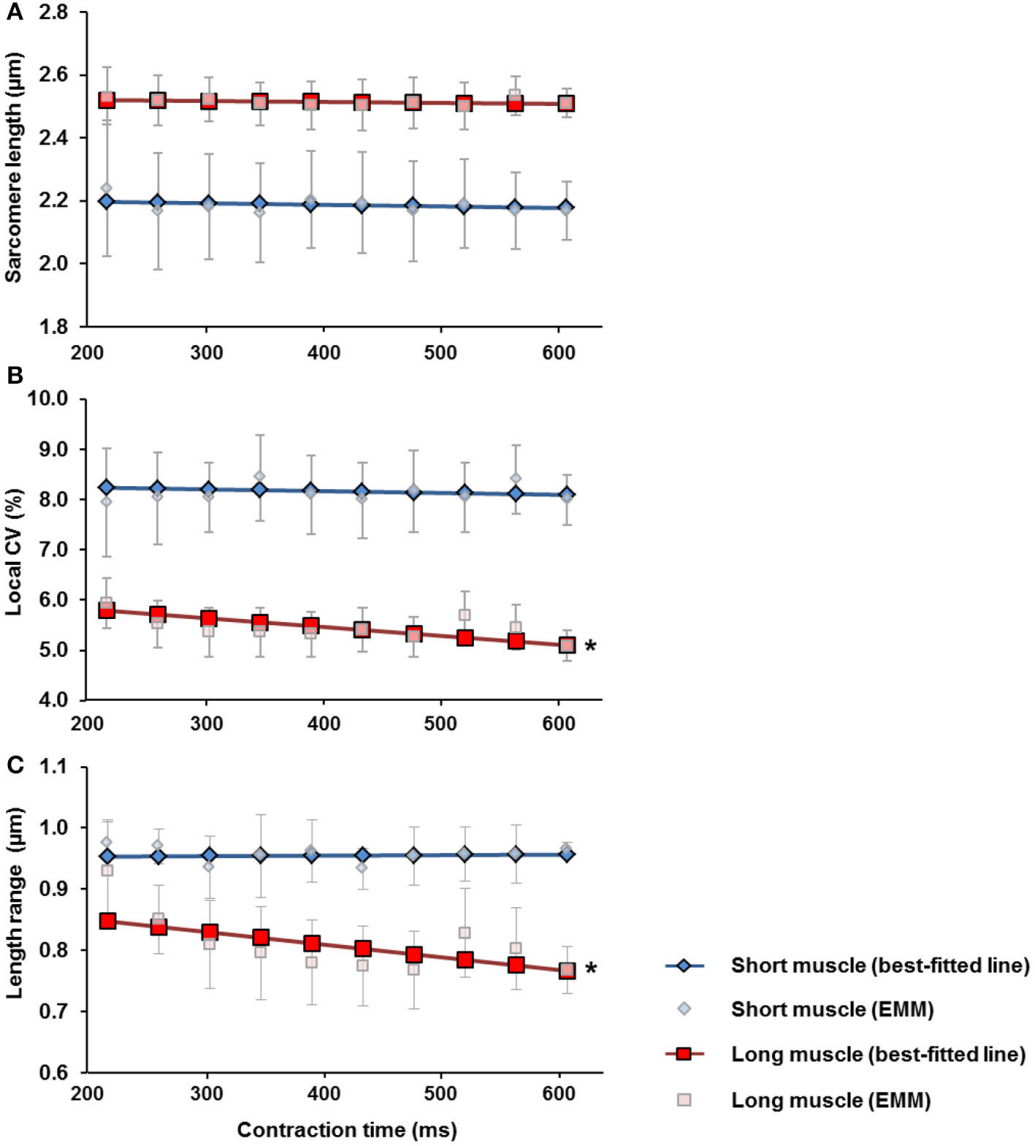
Changes of **(A)** the mean sarcomere length (SLs), **(B)** the local coefficient of variation (CV) of SLs and, **(C)** the SL range, over the 600 ms contraction period measured at the short (blue diamonds) and the long (red squares) muscle lengths. For both muscle lengths, the mean SLs remained constant throughout the contraction period. For the short muscle, the local CV and SL range stayed constant during the 600 ms contraction period. For the long muscle, the local CV and the SL range decreased by ~12% and ~9%, respectively throughout the 600 ms contraction period. ^*^Indicates significant relationship between contraction time and local CV, as well as between contraction time and SL range (*p* < 0.05). EMM-estimated marginal mean.

Mean SLs remained constant throughout the 600 ms contraction period for the short (~2.2 μm) and long muscle length (~2.5 μm; Figure 5A). The local CV and SL range for the activated TA was greater at the short compared to the long muscle length. At the short muscle length, the local CV and SL range remained constant throughout the 600 ms contraction time, while the local CV and SL range at the long muscle length decreased by ~12 and 9%, respectively, during the 600 ms contraction time (Figures 5B,C).

**Figure 6:**
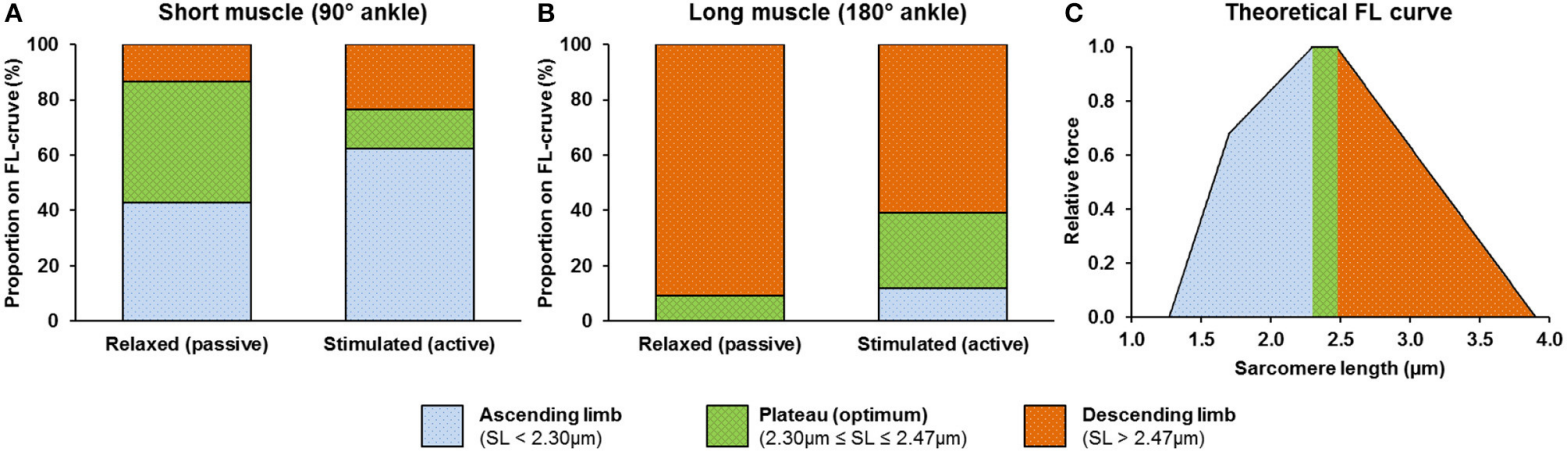
Activation-induced changes in proportion of sarcomeres with lengths that fall onto the ascending limb, plateau region, and descending limb of the theoretical force-length (FL) curve at short **(A)** and long **(B)** muscle lengths. A theoretical FL curve of the mouse TA (Walker and Schrodt, 1974; Herzog et al., 1992; Gokhin et al., 2014) adapted from Gordon et al. (1966) is shown in **(C)**. For the relaxed-short muscle, 44% of the sarcomeres were located on the plateau region of the force-length relationship. For the relaxed-long muscle, 9% of the sarcomeres were located on the plateau region. In the active muscle, 14 and 27% of the sarcomeres were located on the plateau region for the short and long muscle length, respectively.

At the relaxed-short muscle length, 44% of the sarcomeres resided on the plateau of the force-length relationship. This number decreased to 14% during activation (Figure 6A). For the relaxed-long muscle length, 9% of the sarcomeres resided on the plateau of the force-length curve. This number increased to 27% during activation and exceeded the corresponding proportion seen at the short muscle length (Figure 6B).

**Figure 7:**
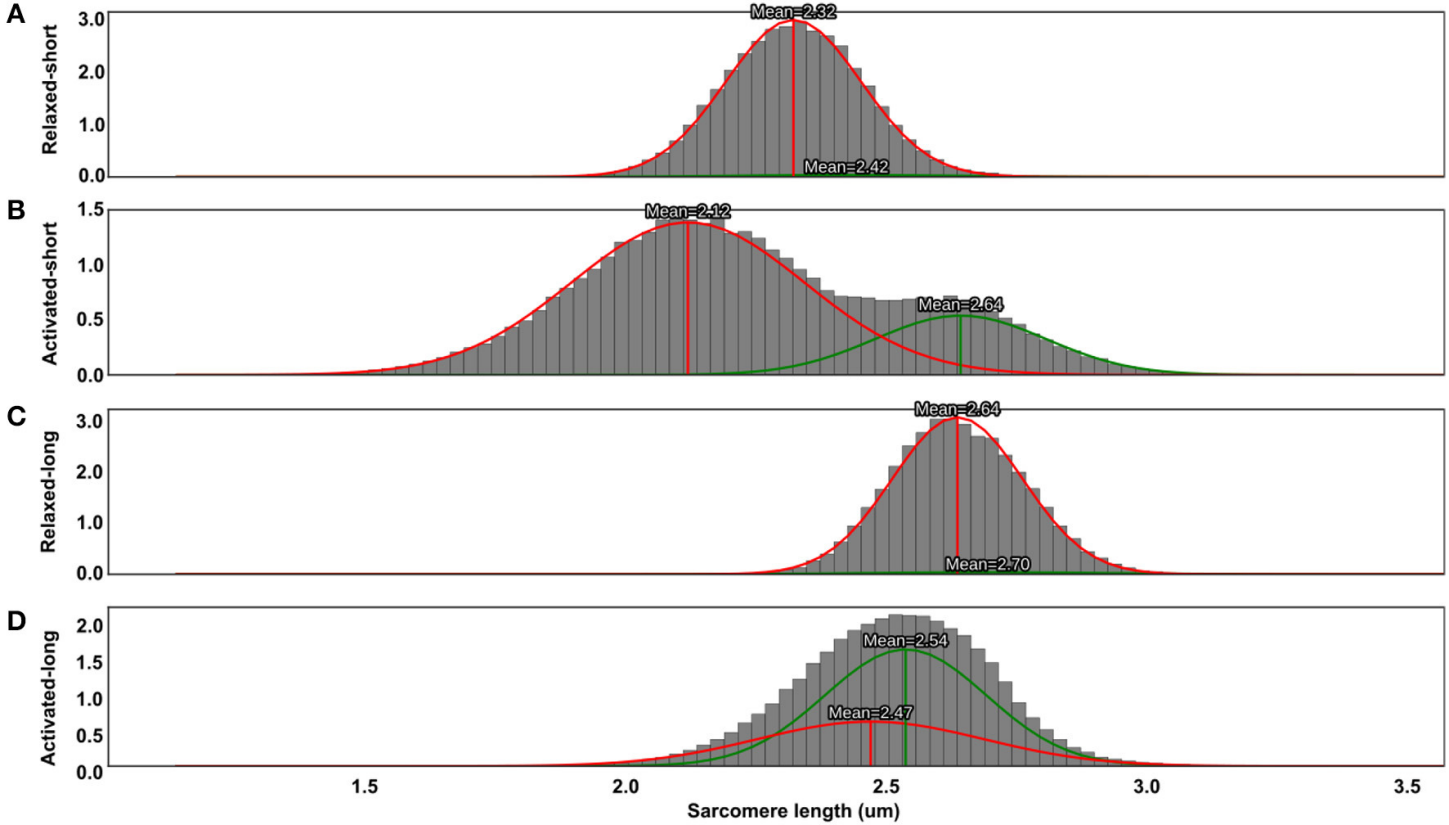
Probability distribution functions (PDFs) of sarcomere lengths pooled from nine tested animals in the relaxed-short **(A)**, activated-short **(B)**, relaxed-long **(C)**, and activated-long **(D)** muscles. Two weighted Gaussian curves (red and green curves) were fitted to the PDFs. Sarcomere lengths in the relaxed muscles had a normal distribution and therefore had only one Gaussian curve with significant weight **(A,C)**. For the activated-short muscles, the distribution of SLs became bimodal, with mean lengths of 2.12 and 2.64 μm **(B)**. However, a similar switch to bimodality in SL distribution was not observed in the activated-long muscle, as the SL distribution became only slightly positively skewed **(D)**. Note that the area under the PDF and the total area under the two weighted Gaussian curves are, respectively, equal to 1.

SLs in the relaxed muscle had a normal distribution (Figures 7A,C). During activation, the SL distribution for the short muscle became bimodal, with mean lengths of 2.12 and 2.64 μm (Figure 7B). These two mean SLs gave rise to a weighted theoretical force of 0.90. The Gaussian-fitted mean SLs yielded a different theoretical force (0.90) compared to the force derived from the simple averaging of the pooled SLs (0.95) shown in Figure 3. In the activated-long muscle, the SL distribution became slightly positively skewed (Figure 7D) with mean SLs of 2.47 and 2.54 μm. These two mean SLs correspond to a weighted theoretical force of 0.97.

**Figure 8:**
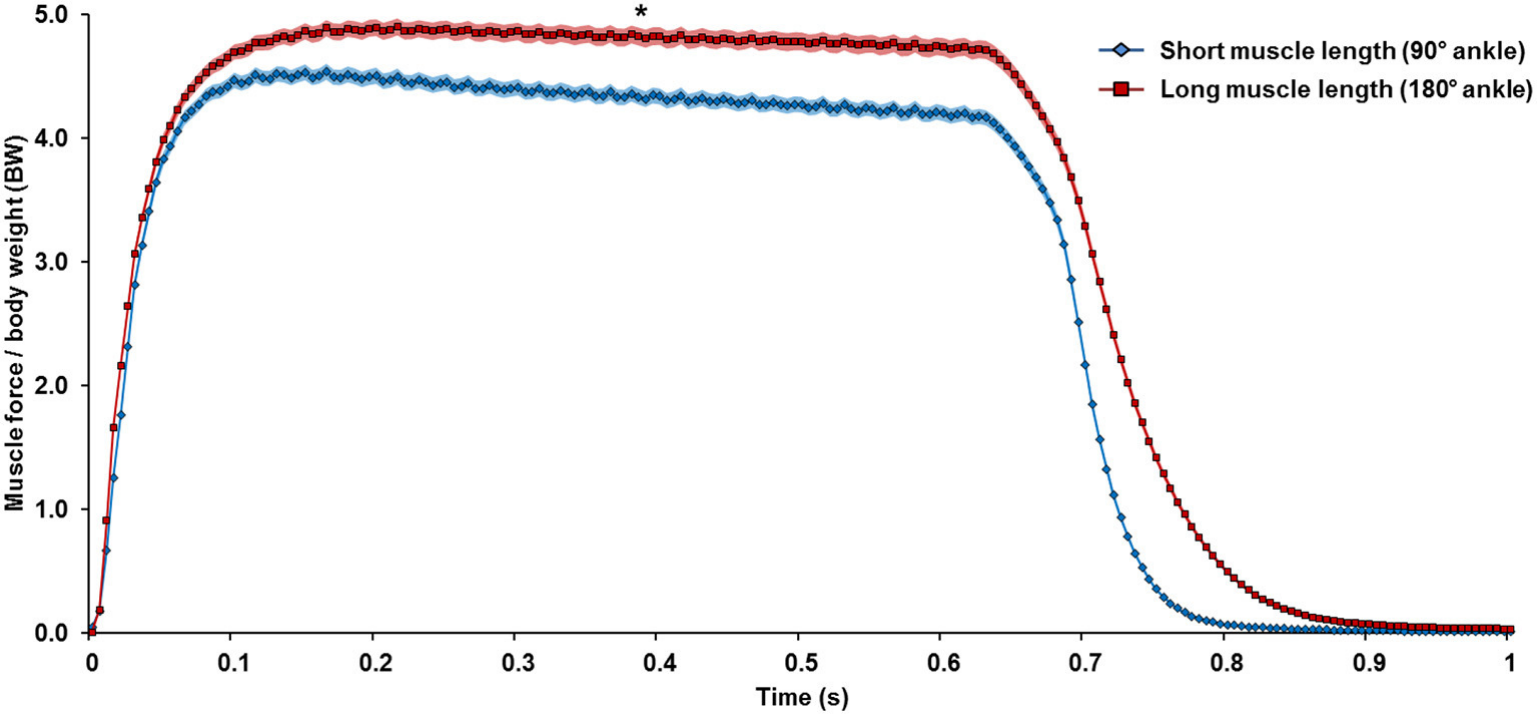
Mean muscle force normalized to body weight (BW) pooled from the trials conducted at the short (118 trials, blue diamond) and long muscle lengths (169 trials, red square) from all nine animals. The color shading indicates the standard error of the mean. On average, the TA muscle generated more force at the long (180° ankle flexion) than the short length (90° ankle flexion). ^*^ Indicates significant differences in force compared with forces measured at the short muscle length (*p* < 0.01).

From the proportion of sarcomeres on the plateau region of the FL curve (Figure 6) and the SL-derived theoretical forces (Figure 7), the muscle is supposed to generate greater forces at the long (180° ankle flexion) than the short (90° ankle flexion) muscle length. Indeed, we measured a 9% greater force at the long compared to the short TA length (Figure 8) at 200 ms of the isometric contraction. Using Equation (2) and the mean SLs derived from the data pooled from nine animals (Figure 7), the force predicted by the mean SLs fell within ±5% of the measured value.

**Figure 9:**
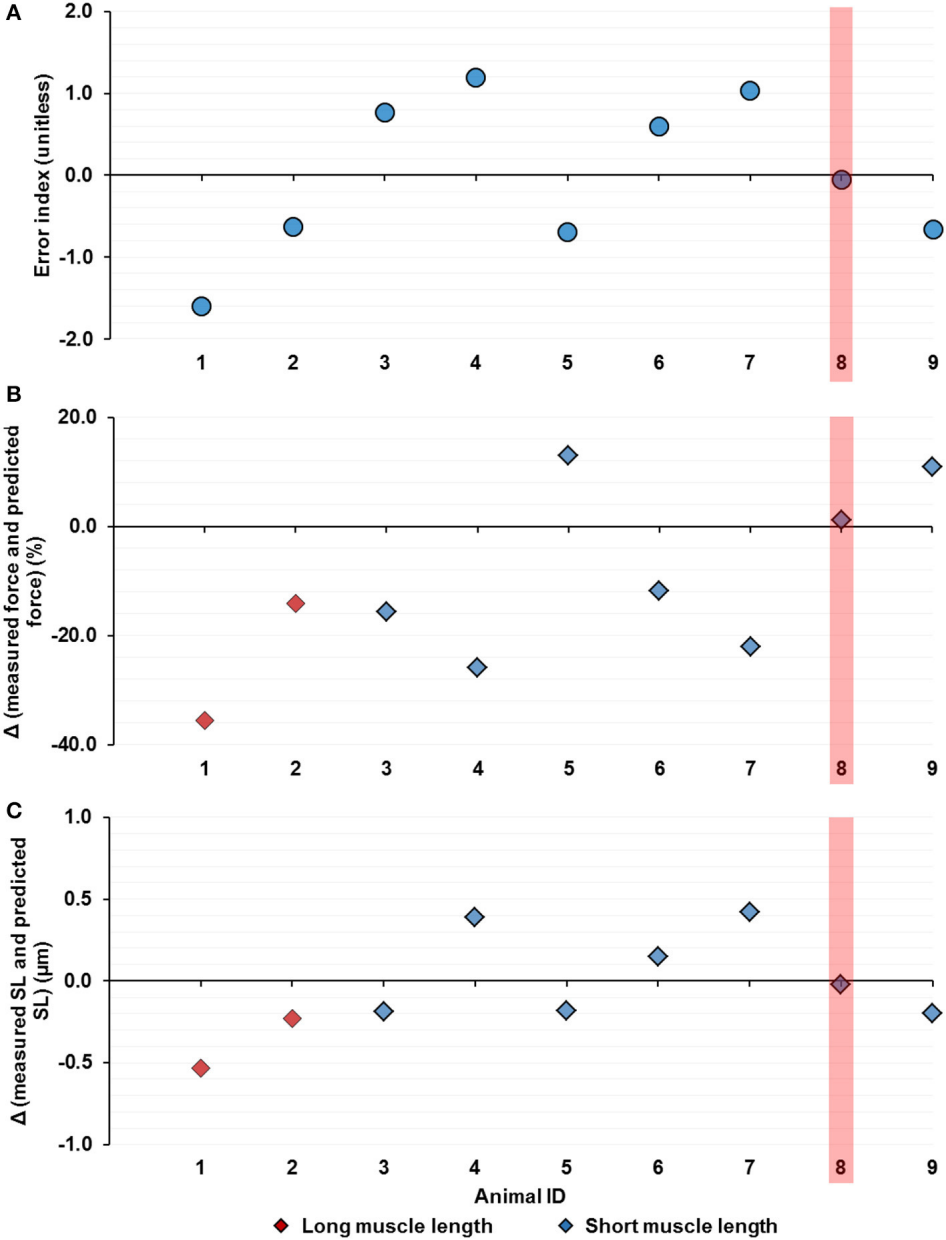
Theoretically predicted forces using the Gaussian-fitted mean sarcomere length (SL) for the individual muscles (N_animal_ = 9) by **(A)** the error index, **(B)** the difference between the measured and the predicted muscle force (normalized by the mean isometric force at the short TA length and measured at 200 ms, see Figure 8) and, **(C)** the difference between the measured and predicted mean SL (see Methods). The successful prediction, which occurs when the predicted value falls within ±5% of the measured value, was found only once out of nine times (highlighted in red). The discrepancy between the predicted and measured values can be as big as ~35% of the isometric force of the short TA **(B)** and ~0.5 μm in absolute SL **(C)**.

Out of the nine tested animals, muscle force was only successfully predicted once to be within ±5% of the measured value (Figure 9). The discrepancy between the predicted and the measured force values and SL was as big as ~35% of the isometric force for the short TA (Figure 9B) and ~0.5 μm in SL, respectively (Figure 9C). Muscle forces and SL PDF for individual animals (*n* = 9) are included in detail in the Supplementary Material S5.

## Discussion

Using an advanced multi-photon microscope and a novel tendon force transducer, *in vivo* individual sarcomere lengths and force in intact whole muscle were measured simultaneously during isometric tetanic contractions. Through a novel imaging protocol, inspired by previous studies on the sarcomere mechanics of the beating heart (Kobirumaki-Shimozawa et al., 2016), sarcomeres from the mid-belly region of the mouse TA were visualized in the relaxed and activated muscle. The primary goal of our study was to investigate the changes in sarcomere length distribution during activation. Sarcomere lengths were measured as the distance between adjacent A-band centroids. In agreement with previous *in vivo* studies (Llewellyn et al., 2008; Cromie et al., 2013; Moo et al., 2016), the dispersion of resting sarcomere lengths (SLs) was found to be ~4% at short and long TA lengths (Figure 4). SL dispersion increased during activation, thereby rejecting our first hypothesis (Figure 4). Increased SL dispersions during activation have been observed in experiments using isolated myofibrils (Telley et al., 2006; Leonard and Herzog, 2010; Rassier and Pavlov, 2010; Johnston et al., 2016), but were not expected in whole muscles where structural proteins and the passive collagen network surrounding fibers, fascicles, and the entire muscle were thought to prevent increases in sarcomere length non-uniformities upon muscle activation. This finding suggests that the presence of stabilizing connective tissues found in the extracellular matrix does not prevent an increase in SL non-uniformity in whole muscle during activation. In other words, the observations of great sarcomere length non-uniformities made in isolated fibers (Huxley and Peachey, 1961; Gordon et al., 1966) and myofibrils (Rassier and Pavlov, 2010; Johnston et al., 2016) also occur in entire, *in vivo* muscle preparations. Nevertheless, the connective tissues in the intact muscle likely attenuate the SL dispersion during activation as the high CV of 20–30% found in active rabbit psoas myofibrils (Rassier and Pavlov, 2010; Johnston et al., 2016) were reduced to 6–8% CV in the intact mouse TA (Figure 4).

We found that the increase in SL dispersion was more pronounced (~3 times higher) at the short compared to the long TA length. Passive tension in mouse TA starts developing at sarcomere lengths in excess of about 2.5 μm (Wood et al., 2014). Since the mean SL in the relaxed-long muscle was 2.6 μm (Figures 3A, 7), passive structural muscle elements were likely stretched at this length prior to activation and helped stabilize the A-bands during activation (Horowits and Podolsky, 1987). This is not the case for sarcomeres at the short muscle length (SL of ~2.3 μm; Figures 3A, 7) where the passive structural elements were likely slack prior to activation, possibly allowing for increased A-band shifts and increased SL non-uniformities upon muscle activation (Horowits and Podolsky, 1987). Another interesting finding was that the SLs at the long muscle length became more uniform during the 600 ms isometric contraction (Figures 5B,C). Future studies should investigate the mechanism and functional implication of this increased uniformity in SL at long muscle length.

The biggest CV measured in this study was merely ~8% (Figure 4B), which seems small but is obtained from a large number of individual sarcomeres. A better representation may be the range of SLs in a small muscle region (159 × 2.8 μm^2^) of no more than 30 serial sarcomeres, which was typically ~1 μm for the active TA (Figure 3D). This difference in SL corresponds to more than 40% of the optimal sarcomere length in mouse skeletal muscle. On the descending limb, a SL difference of 1 μm would theoretically correspond to a difference of 70% of the maximal isometric force, and on the ascending limb of the force-length curve, would be the difference between zero force and maximal force. Therefore, the secondary goal of this study was to evaluate the functional implications of this increased SL non-uniformity during activation. Specifically, we were interested if the mean SL, measured in both relaxed and activated states, is a good predictor of the force in the whole muscle. In the relaxed-short TA, 44% of all sarcomeres were located on the plateau of the force-length curve, while in the relaxed-long TA, 9% of all sarcomeres were on the plateau region (Figures 6A,B). The Gaussian-fitted mean SLs were 2.32 and 2.64 μm at the short and long MTU length, which corresponds to theoretical forces of 1.0 and 0.88, respectively (Figure 7). Based on these resting SLs, a muscle at the short length should produce ~14% more force than the same muscle at the long length. However, the experimentally measured forces showed the opposite result: the muscle at the long length generated ~9% greater force than that measured at the short length (Figure 8). If the mean SLs measured during activation were used instead, the resulting theoretical forces derived from the FL curve were within ±5% of the actual force measurements (see “Results”). Assessment of the proportions of SLs on the plateau regions of the FL curve in the activated muscle (Figures 6A,B) also suggested that the muscle should generate higher force at the long (27% sarcomeres on the plateau) rather than the short TA length (14% sarcomeres on the plateau). Therefore, we conclude that the active mean SL is a better predictor of muscle force than the resting mean SL. This finding may appear obvious, but in many studies, sarcomere lengths of relaxed muscles have been taken as indicators of the functional capacity of muscles *in vivo* (Rack and Westbury, 1969; Wickiewicz et al., 1983; Lieber and Boakes, 1988; Lutz and Rome, 1994), which seems inappropriate based on the current findings.

Encouraged by the fact that the muscle force was successfully predicted using the mean SL of the pooled data set, we went on to investigate if the Gaussian-fitted mean SL can predict force in the TAs of the individual animals. To our surprise, only in one out of nine cases did the predicted force fall within ±5% of the true values measured experimentally. The differences between the predicted and the measured forces ranged from about 11–35% of the maximal isometric force at the short TA length (Figure 9B). In terms of the SLs, the predicted values were ~0.2–0.5 μm off from the measured mean sarcomere length values (Figure 9C). In an intact whole muscle, the muscle architecture and the surrounding connective tissue likely alter the force transmission from the myofibril level to the whole muscle, making it unreliable to predict muscle force using the mean SL. Furthermore, we showed previously that sarcomere lengths vary dramatically for neighboring sarcomeres within the same region of the muscle, and that the mean sarcomere lengths vary substantially from one location of the muscle to the next (Moo et al., 2016). To complicate matters even further, sarcomere length changes associated with muscle length changes also differ between muscle locations (Moo et al., 2016). Therefore, the mean sarcomere lengths measured at one point and at one length of the muscle is likely not representative of the true mean sarcomere length, and thus might cause the erroneous force predictions observed here.

There are limitations in this study that need careful consideration when interpreting our results. First, a few layers of skin and fascia covering the TA were removed for optimal SHG imaging. However, as no visible changes were observed in the structure of the muscle (Figure 1), we assume that the sarcomere length measurements obtained in this manner are identical to those that would be obtained in the fully intact muscle. Second, the imaging protocol used in this study, though novel, does not allow for tracking of single myofibrils during activation. Instead, the muscle images acquired in the relaxed and activated states represent sarcomeres from the same region in the muscle (within an area of ~50 μm in diameter). Finally, due to the poor signal-to-noise ratio for the deep tissue images, sarcomere images were obtained from a tissue depth of up to ~100 μm only. Despite the aforementioned limitations, our study provides novel insight into sarcomere dynamics and the relationship between SL and force in intact muscles. Future studies should focus on studying the relationship between mean SL measured during activation and the resulting muscle force at multiple muscle lengths with smaller length increment to provide additional support for the current findings. As the sarcomeres across a whole muscle have variable lengths (Moo et al., 2016; O'Connor et al., 2016), future investigations should also study sarcomeres at different anatomical locations, for example at the distal TA, by following the current experimental protocols.

## Conclusion

The results of this study warrant the following conclusions: (i) sarcomere length dispersion (non-uniformities) increases substantially from the relaxed (passive) to the activated state and reaches average differences of ~1.0 μm in a small segment of muscle comprised of ~30 consecutive sarcomeres; These differences in sarcomere length are associated with theoretical force differences of 70–100% of the maximal isometric force; and (ii) the mean sarcomere lengths measured at a single location in the relaxed muscle are poor indicators of the force potential of the muscle. Mean sarcomere lengths in the activated muscle are better predictors of force, but can still be erroneous by up to 35% under the present study conditions, which are optimal and probably cannot be replicated in a human study at this time.

## Author contributions

Substantial contributions to the conception or design of the work; or the acquisition, analysis, or interpretation of data for the work: EM, TL, and WH. Drafting the work or revising it critically for important intellectual content: EM and WH. Final approval of the version to be published: EM, TL, and WH. Agreement to be accountable for all aspects of the work in ensuring that questions related to the accuracy or integrity of any part of the work are appropriately investigated and resolved: EM, TL, and WH.

### Conflict of interest statement

The authors declare that the research was conducted in the absence of any commercial or financial relationships that could be construed as a potential conflict of interest.
