# Supplementary material for: In Vivo Sarcomere Lengths Become More Non-uniform upon Activation in Intact Whole Muscle
Source: Front Physiol. 2017 Dec 7;8:1015. doi: 10.3389/fphys.2017.01015 (PMC5725405; doi:10.3389/fphys.2017.01015)
Supplement: Supplementary file 1 [file Table1.pdf]

Supplementary Material

In Vivo Sarcomere Lengths Become More Non-Uniform Upon Activation in Intact Whole Muscle

Eng Kuan Moo[1], Timothy R. Leonard[1], Walter Herzog[1*]

[1]Human Performance Laboratory, Faculty of Kinesiology, University of Calgary, Calgary, Alberta, Canada

*** Correspondence:**
Name: Walter Herzog
Address: Human Performance Laboratory, University of Calgary, 2500 University Drive N.W., Calgary, Alberta, T2N 1N4, Canada.
Tel: +1 403 220 8525; E-mail: wherzog@ucalgary.ca

S1: E-shaped buckle-type tendon force transducer – design and calibration

A custom-made buckled-type tendon force transducer was built and miniaturized based on the design of Herzog *et al*. (1993) and the conceptual idea of Walmsley *et al.* (1978). The tendon force transducer was made of an E-shaped stainless steel (316 alloy) base element instrumented with two strain gauges (model ESU-60-350, Entran Devices Inc., NJ, USA) in a half Wheatstone bridge configuration (Fig. R1). The distal tendon of the mouse tibialis anterior (TA) was woven through the three arms of the transducer and was secured to the transducer by closing the open sides of the E-shaped element with sutures.

In order to calibrate the tendon force transducer, the TA distal tendon attached to the tarsal bone was dissected at the distal end and known weights were hung from the free end of the tendon. The resulting voltage signals from the force transducer showed a virtually perfect (r^2=0.99) linear correlation with the applied force (Fig. R2).

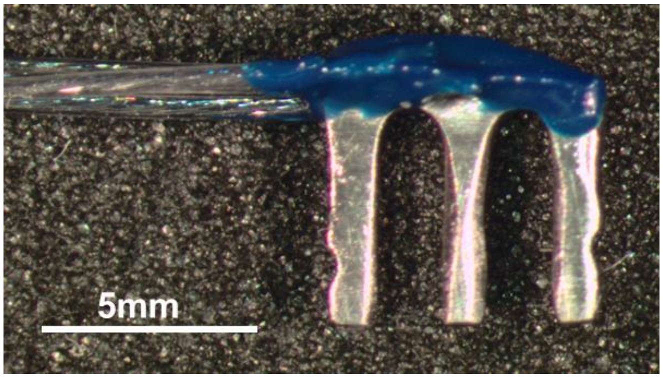

Fig. R1. The miniature tendon force transducer used to measure the forces in the mouse TA

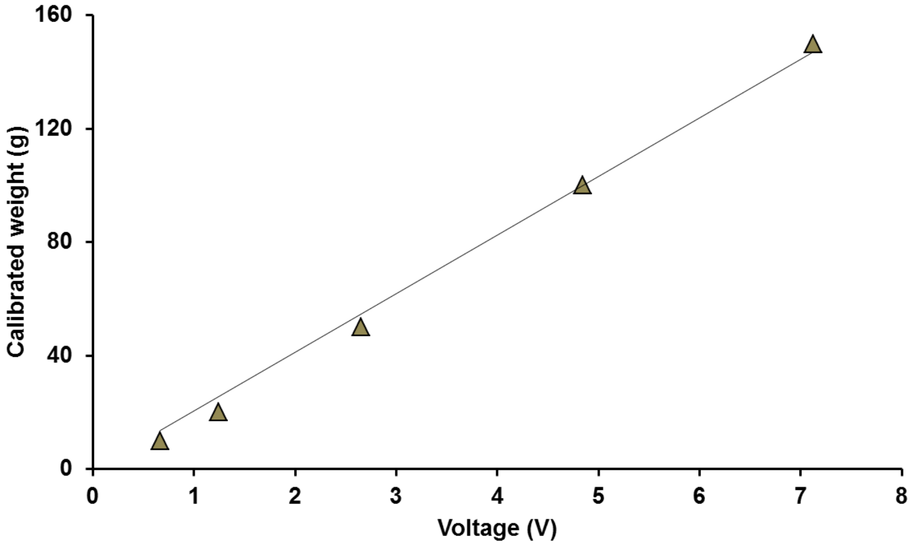

Fig. R2. The calibration curve of the tendon force transducer showed a strong linear relationship between the known weights and the resulting voltage (r^2=0.99).

S2: Determination of the scanning regions in the muscle under relaxed and activated conditions

In order to estimate the total displacement of the site of sarcomere length measurement during muscle activation, two fluorescent markers separated by ~1mm along the muscle longitudinal axis were attached to the mid-belly of the muscle by using a 100µm-diameter glass tip attached to a 3-axes linear micro-manipulator (Newport Corp., CA, USA). The displacements of these two fluorescent markers (d_1, d_2, see Fig. R3) were carefully measured by observing through the eyepiece of a multi-photon excitation microscope (FVMPE-RS model, Olympus, Tokyo, Japan) under fluorescent light.

A scanning area was selected between the two fluorescent markers and the TA muscle was imaged in its relaxed state. The displacement of the scanning area ($d_{scanning\_area}$) during muscle activation was determined by linear interpolation of the displacements of the two fluorescent markers (d_1, d_2). A sub-micron precision, motorized microscope stage (Prior Scientific, MA, USA) on which the animal sat was then used to displace the TA muscle by '$d_{scanning\_area}$' in the horizontal plane (XY-plane). The TA muscle was then supra-maximally stimulated and imaged simultaneously at this XY-coordinate in order to image sarcomeres from the same region (within an area of ~50µm diameter) as in the relaxed muscle.

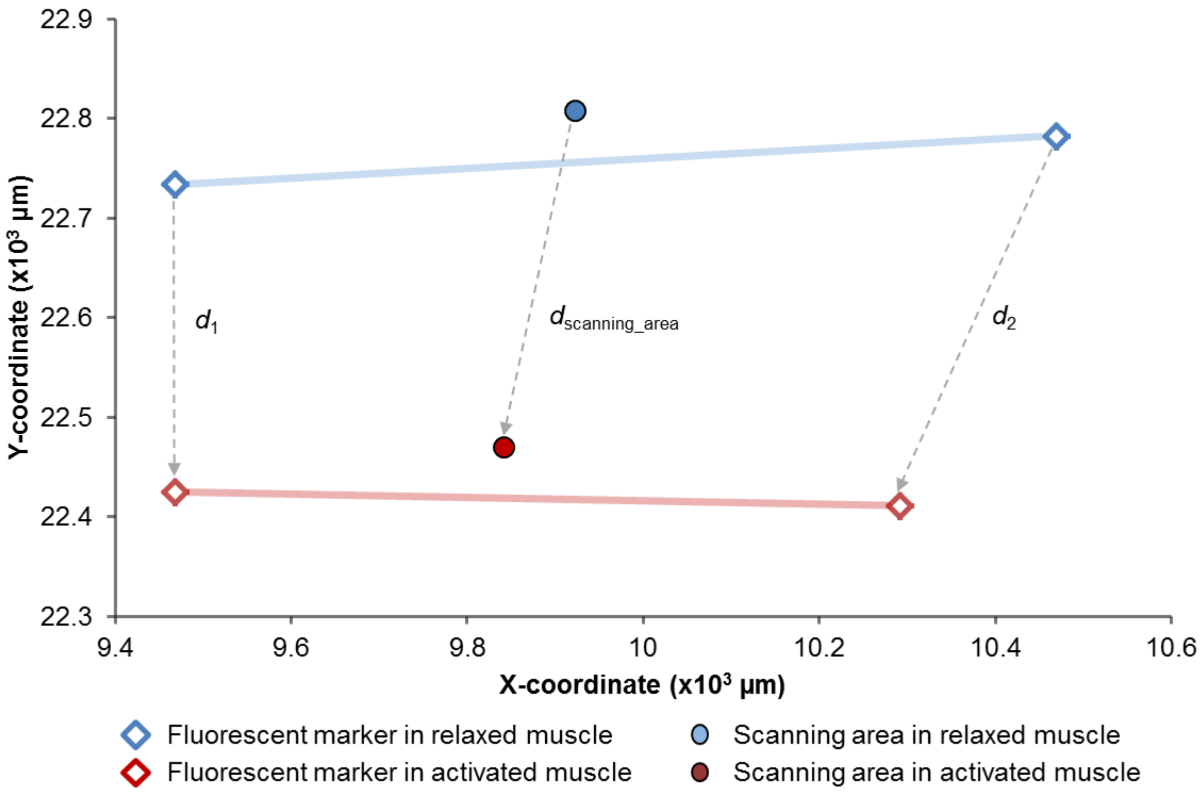

Fig. R3. XY-coordinate map on a sub-micron precision motorized microscope stage showing the displacements of the fluorescent markers (diamonds in blue and red) on the mouse TA during activation. The displacements of these two fluorescent markers were then used to determine the displacement of the scanning area (circles in blue and red) during activation to allow for imaging of sarcomeres from the same location in the relaxed and active state.

S3: Correction for out-of-plane orientation in sarcomere length (SL) measurements

Using the through-thickness muscle image in the XZ-plane (Fig. R4A), the orientation of the epimysium ($\theta_{surface}$) and the angle between the epimysium and the sarcomeric A-bands ($\theta_{A\text{-}band}$) were determined (Fig. R4B). As the time-series planar images of sarcomeres were taken in the horizontal plane, the SL measured ($SL_{measured}$) from this images were corrected for out-of-plane projection using the sine rule as shown in Eq. (R1) and Eq. (R2) to obtain SL along the epimysium ($SL_{corrected}$):

$$\frac{SL_{corrected}}{\sin(180-\theta_{surface}-\theta_{A-band})} = \frac{SL_{measured}}{\sin(\theta_{A-band})} \tag{R1}$$

$$SL_{corrected} = \frac{\sin(180-\theta_{surface}-\theta_{A-band})}{\sin(\theta_{A-band})} \times SL_{measured} \tag{R2}$$

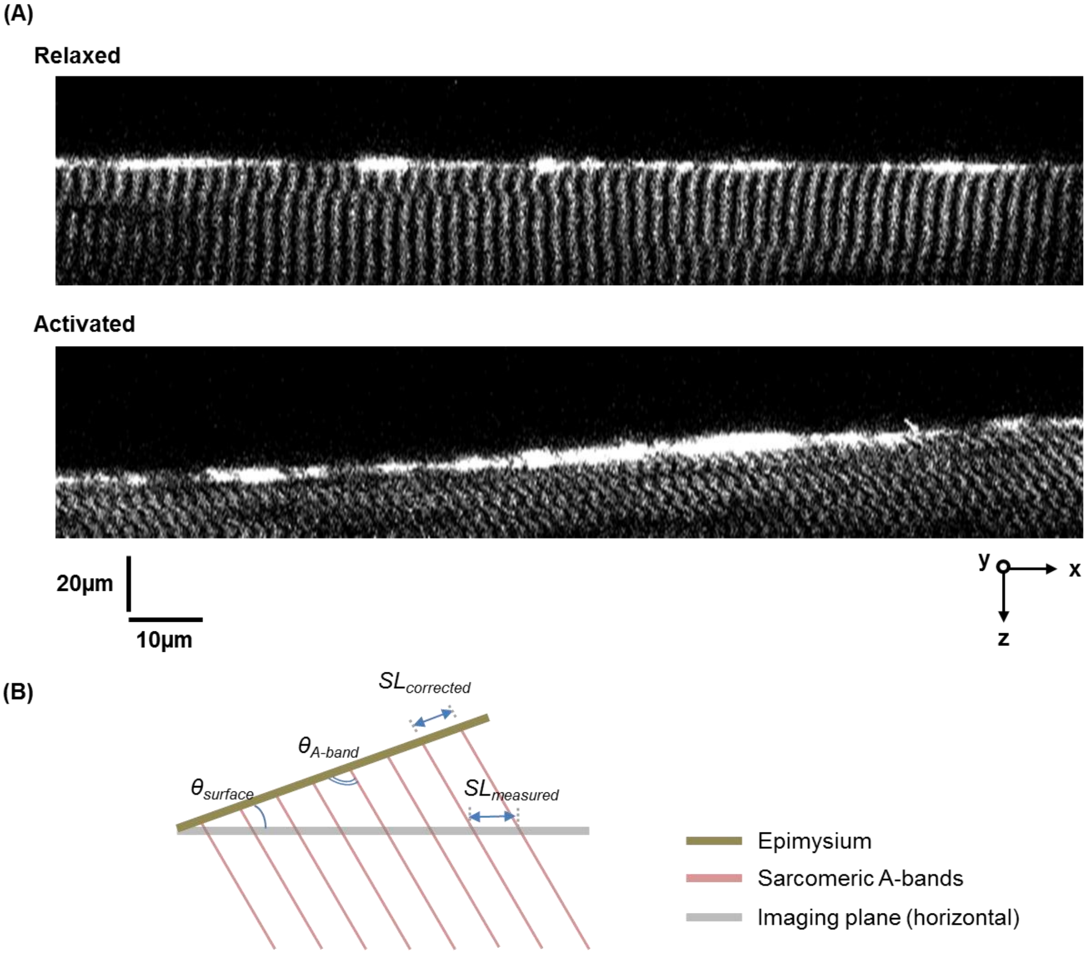

Fig. R4. (A) Through-thickness muscle taken in the XZ-plane showing the orientations of the epimysium (top layer) and the sarcomeric A-bands (white bands) in the relaxed and activated muscle. (B) Schematic diagram showing the angles ($\theta_{surface}$ and $\theta_{A\text{-}band}$) measured from the through-thickness images in order to correct for the out-of-plane projection and obtain sarcomere lengths along the epimysium ($SL_{corrected}$).

S4: Muscle forces and the corresponding sarcomere length (SL) probability distribution functions (PDF) for the nine individual animals

Animal ID: 1

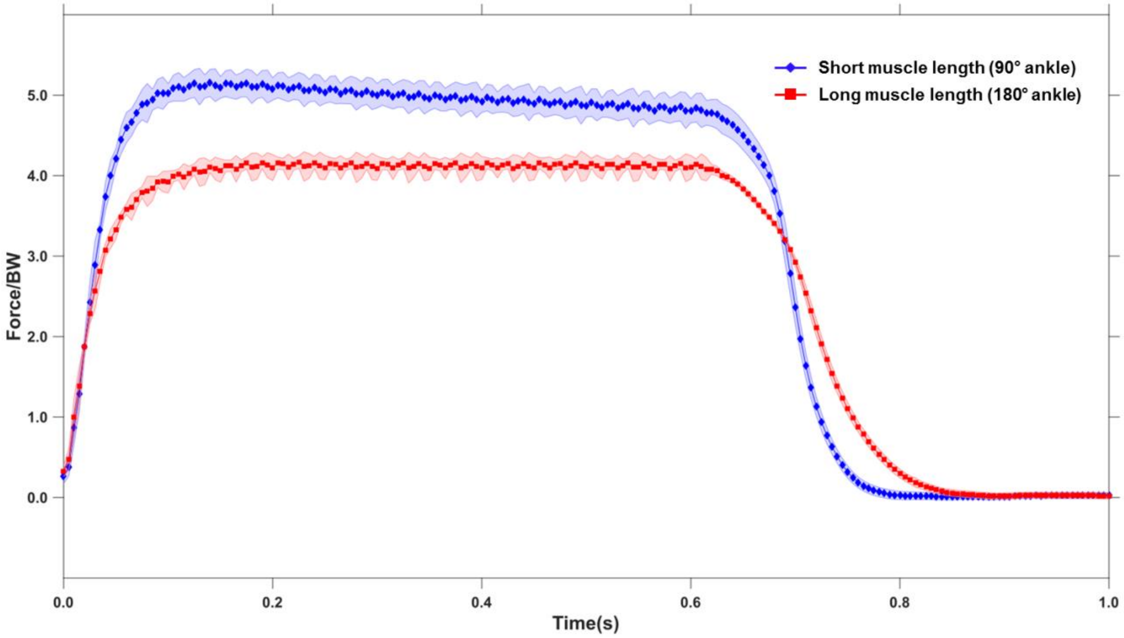

Fig. R5. Forces recorded at short and long muscle lengths for 'animal 1'.

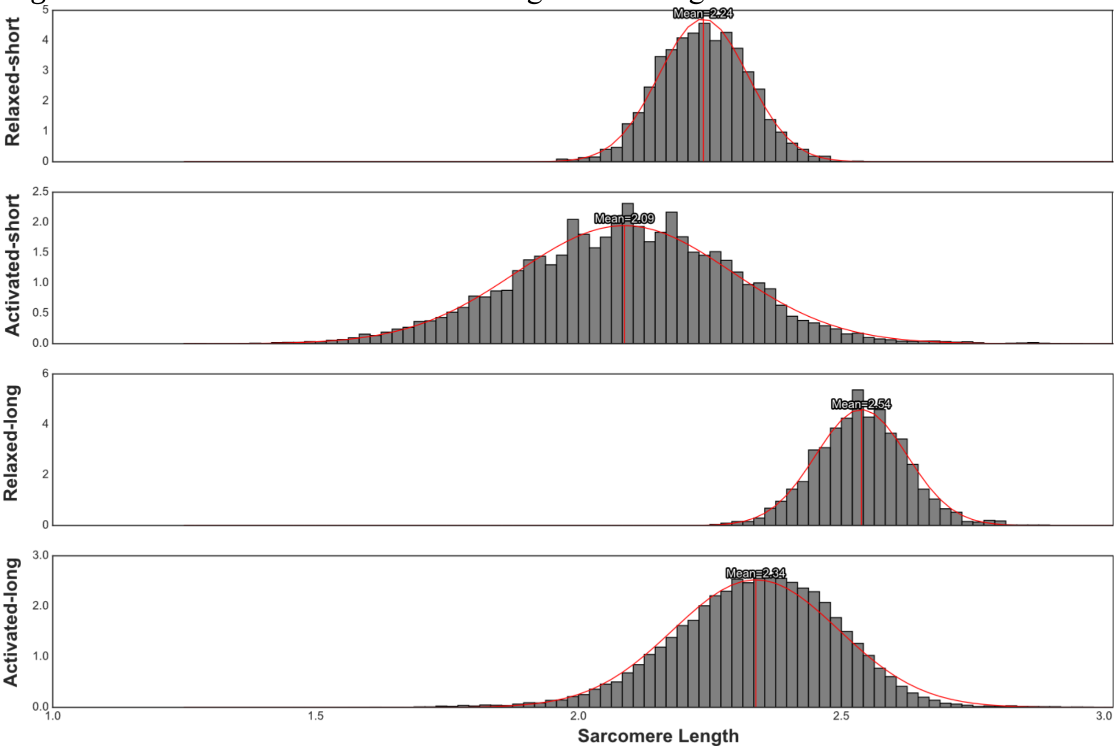

Fig. R6. SL PDF measured at short and long muscle lengths under relaxed and activated conditions for 'animal 1'.

Animal ID: 2

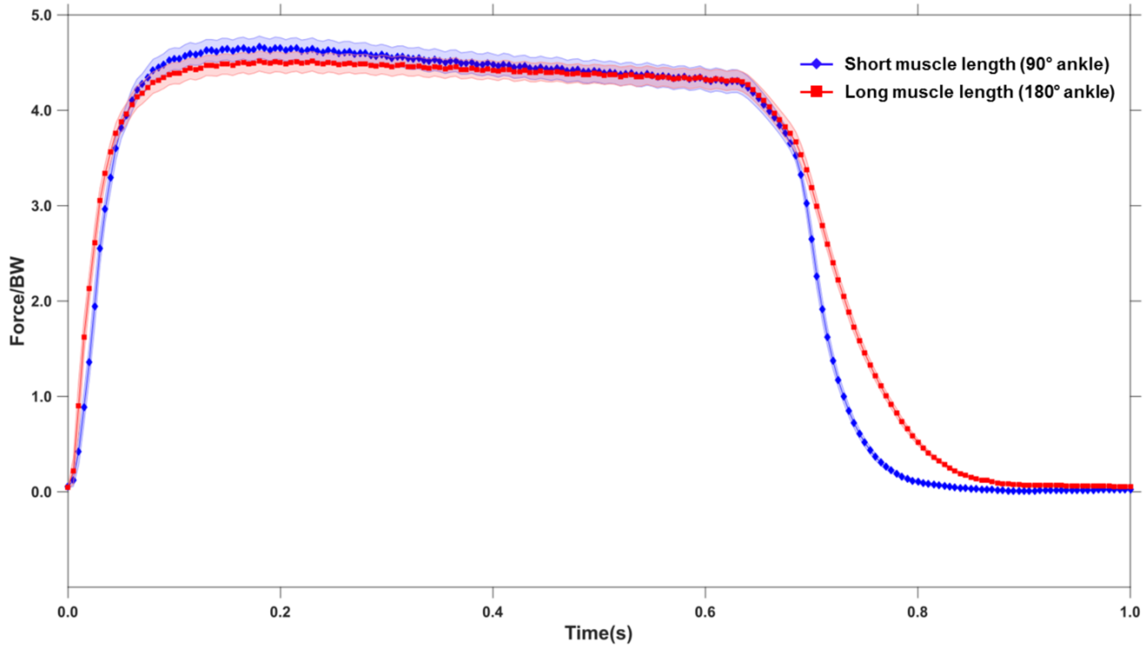

Fig. R7. Forces recorded at short and long muscle lengths for ‘animal 2’.

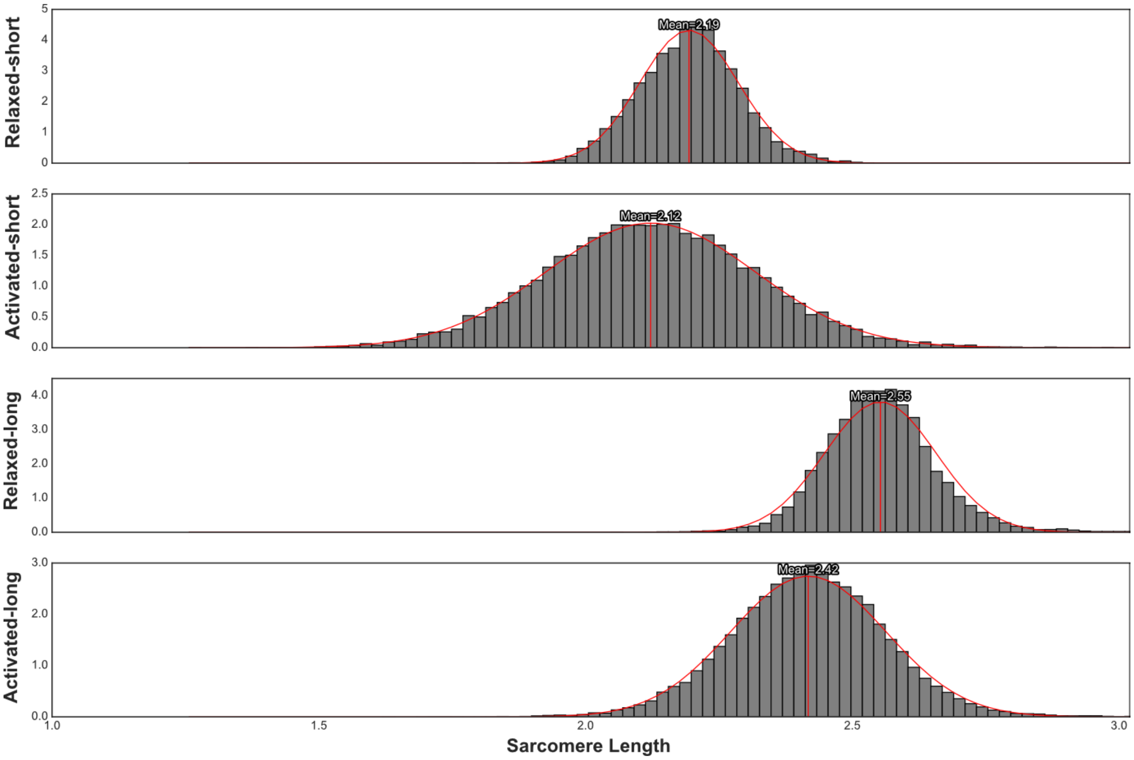

Fig. R8. SL PDF measured at short and long muscle lengths under relaxed and activated conditions for ‘animal 2’.

Animal ID: 3

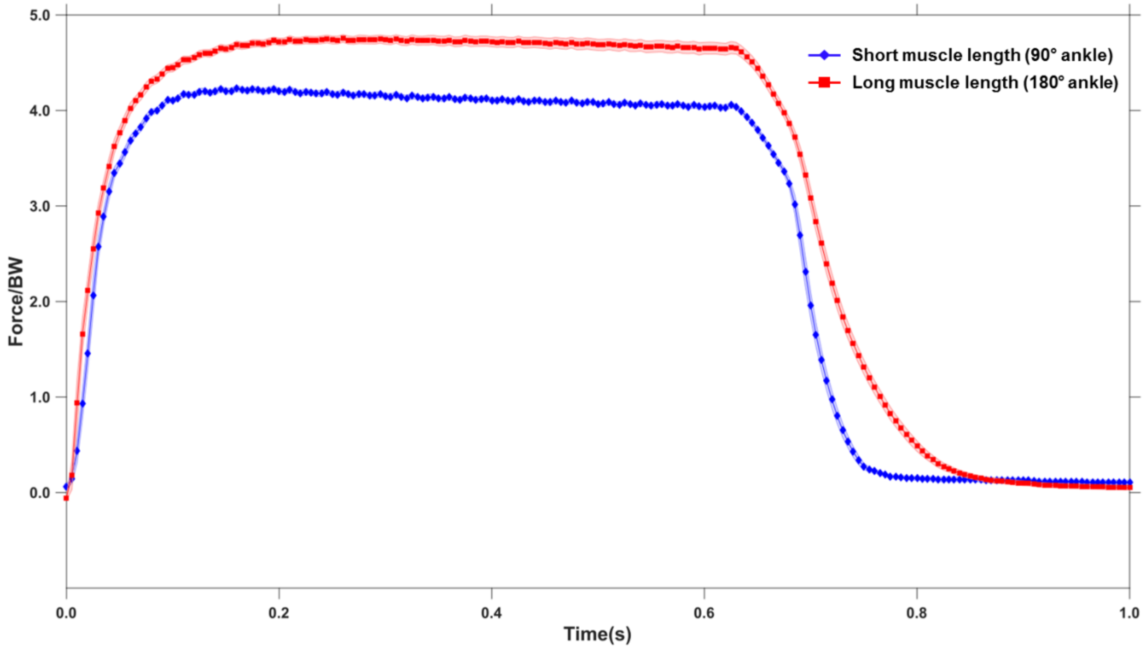

Fig. R9. Forces recorded at short and long muscle lengths for ‘animal 3’.

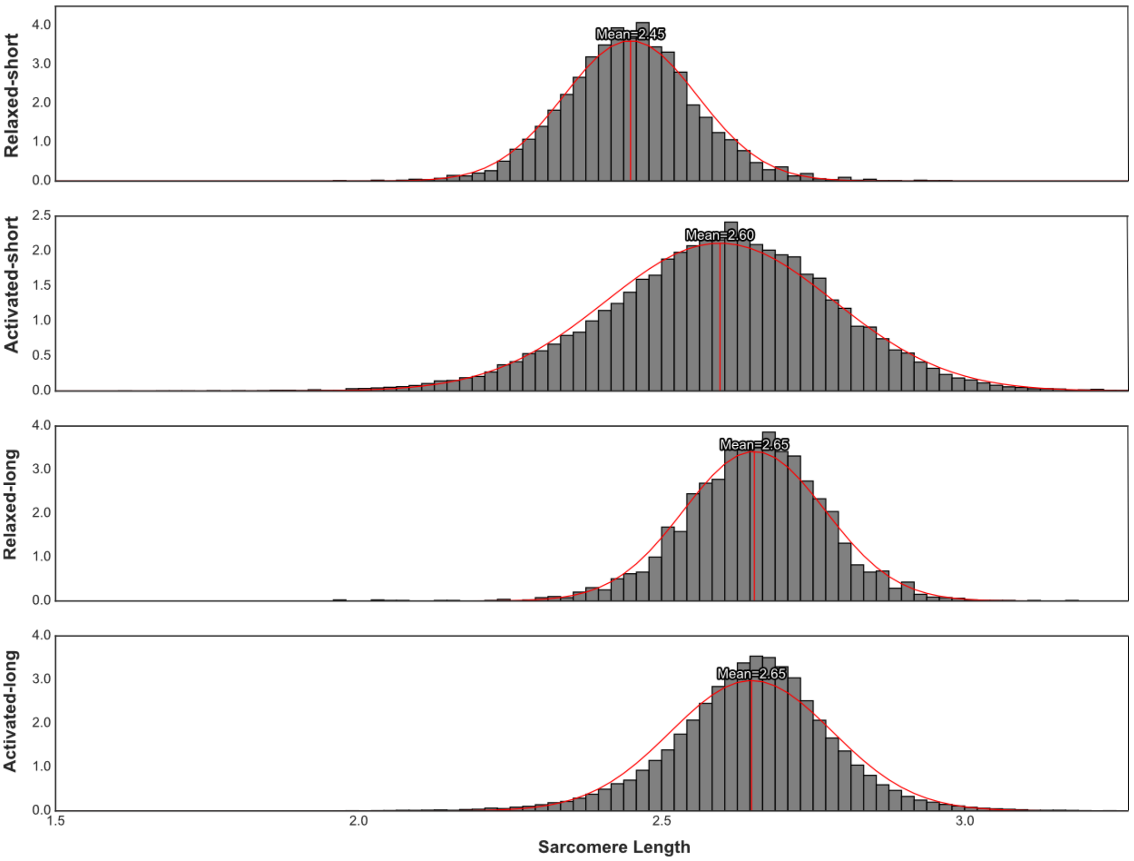

Fig. R10. SL PDF measured at short and long muscle lengths under relaxed and activated conditions for ‘animal 3’.

Animal ID: 4

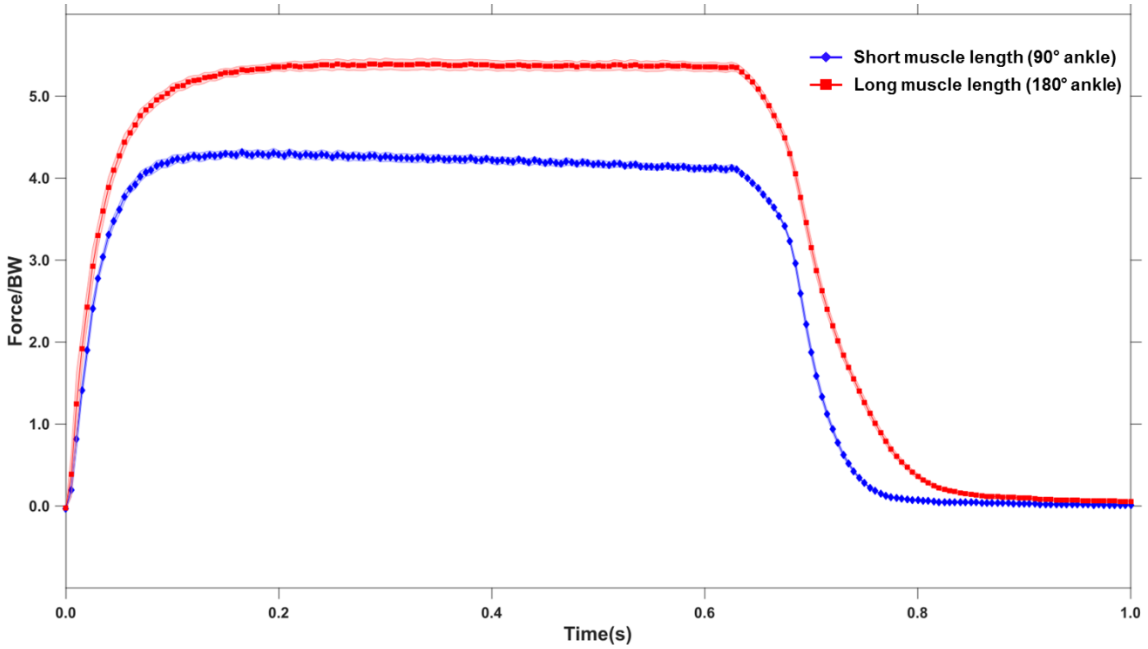

Fig. R11. Forces recorded at short and long muscle lengths for ‘animal 4’.

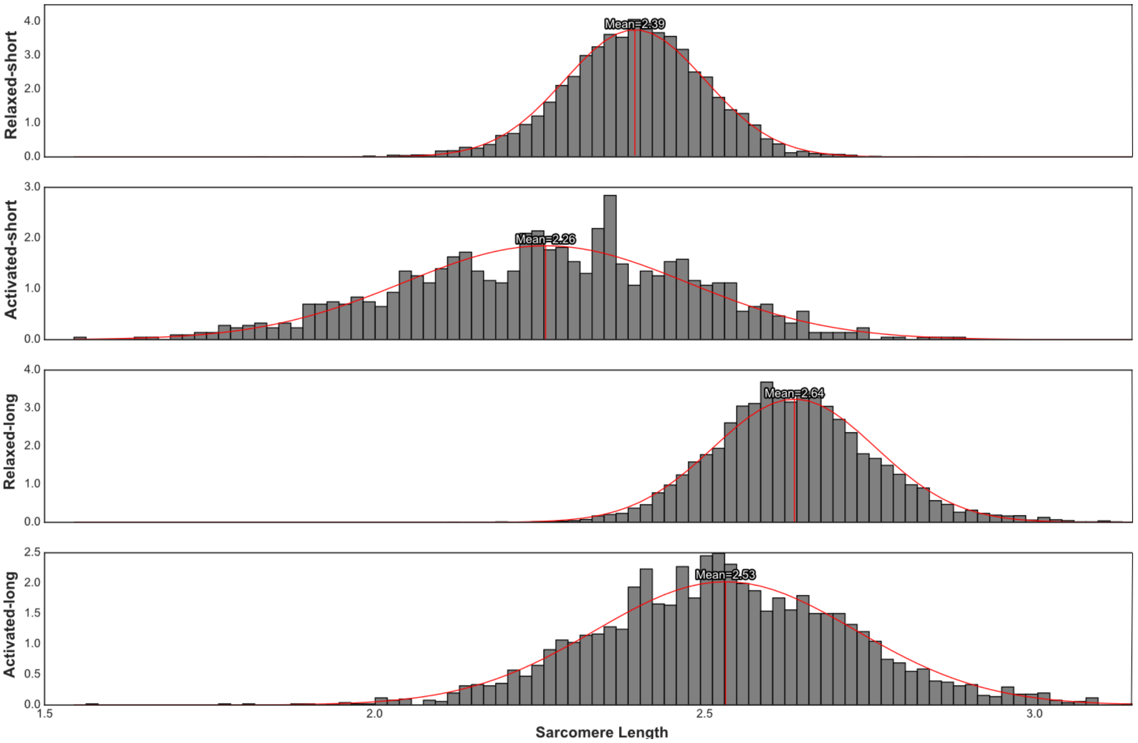

Fig. R12. SL PDF measured at short and long muscle lengths under relaxed and activated conditions for ‘animal 4’.

Animal ID: 5

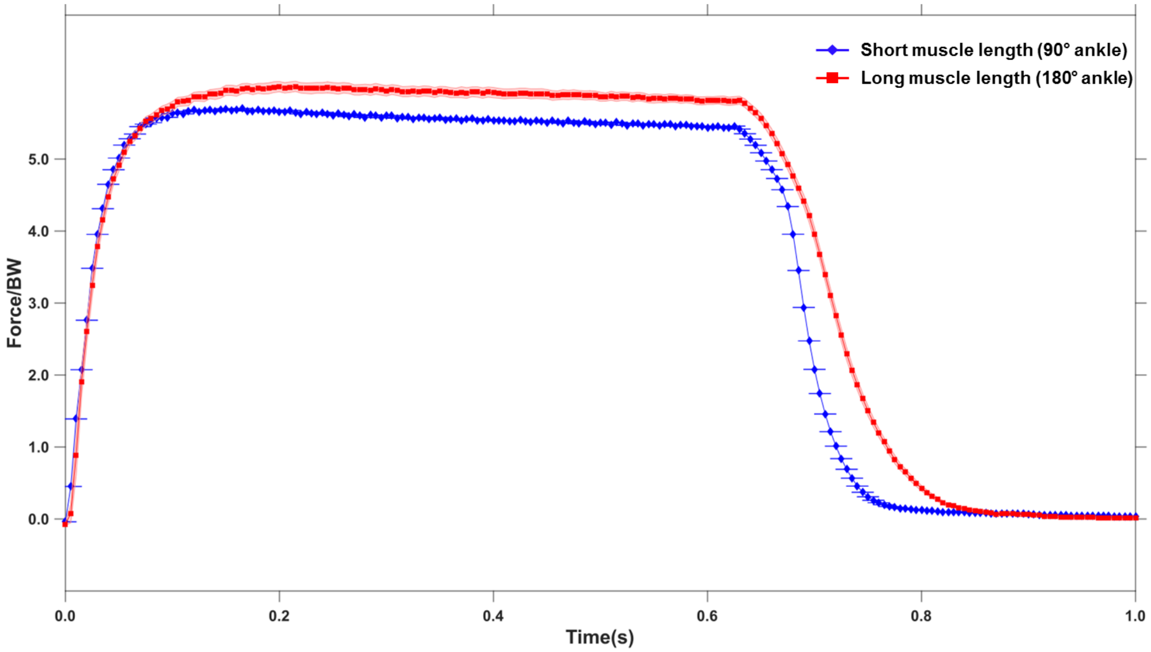

Fig. R13. Forces recorded at short and long muscle lengths for 'animal 5'.

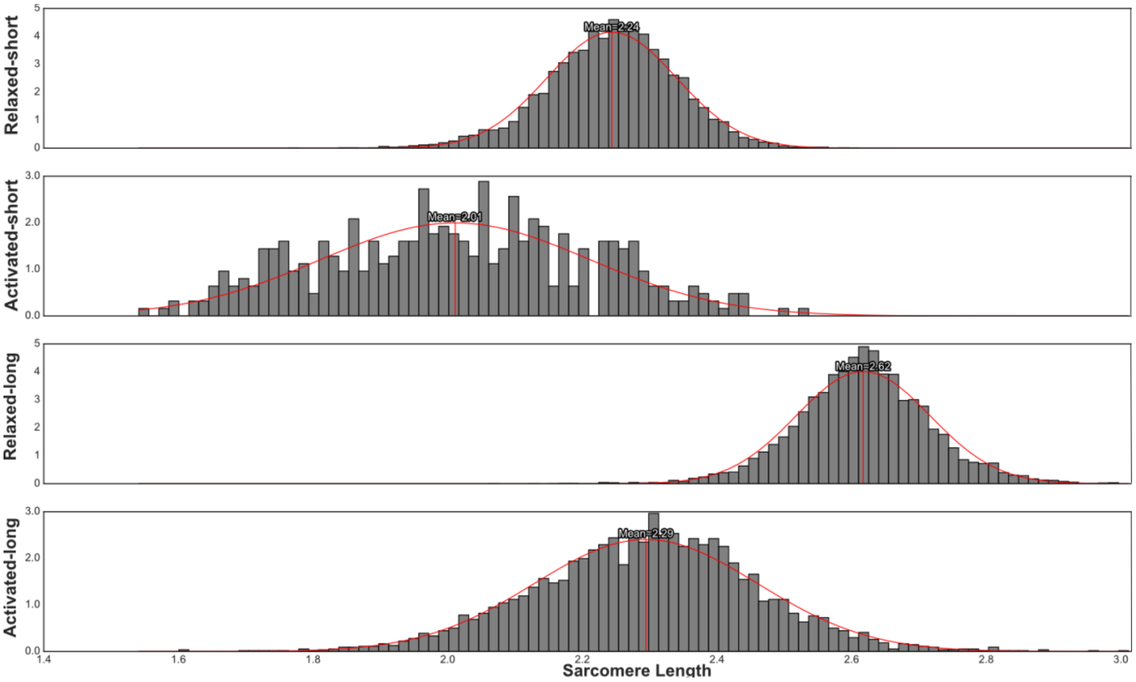

Fig. R14. SL PDF measured at short and long muscle lengths under relaxed and activated conditions for 'animal 5'.

Animal ID: 6

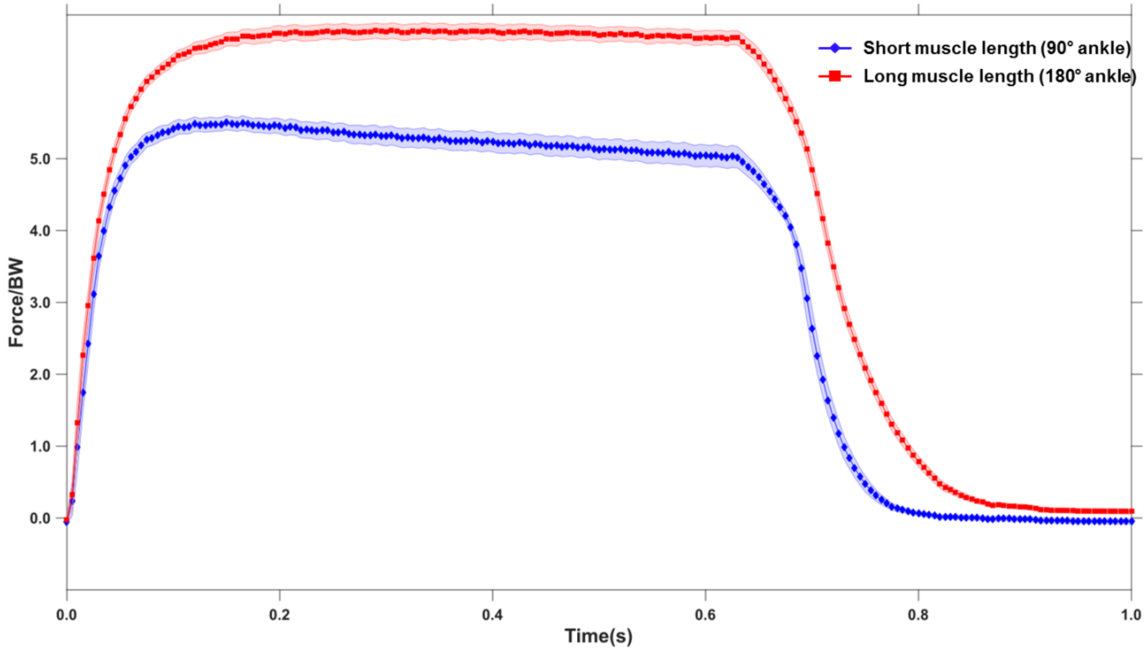

Fig. R15. Forces recorded at short and long muscle lengths for ‘animal 6’.

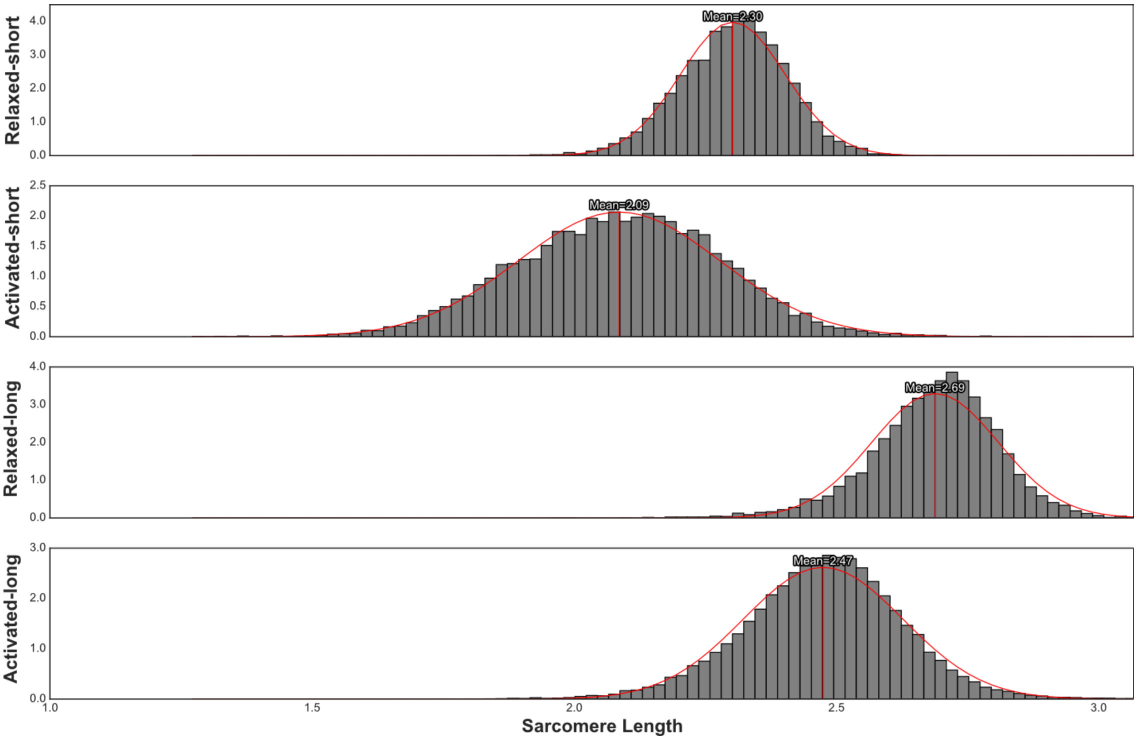

Fig. R16. SL PDF measured at short and long muscle lengths under relaxed and activated conditions for ‘animal 6’.

Animal ID: 7

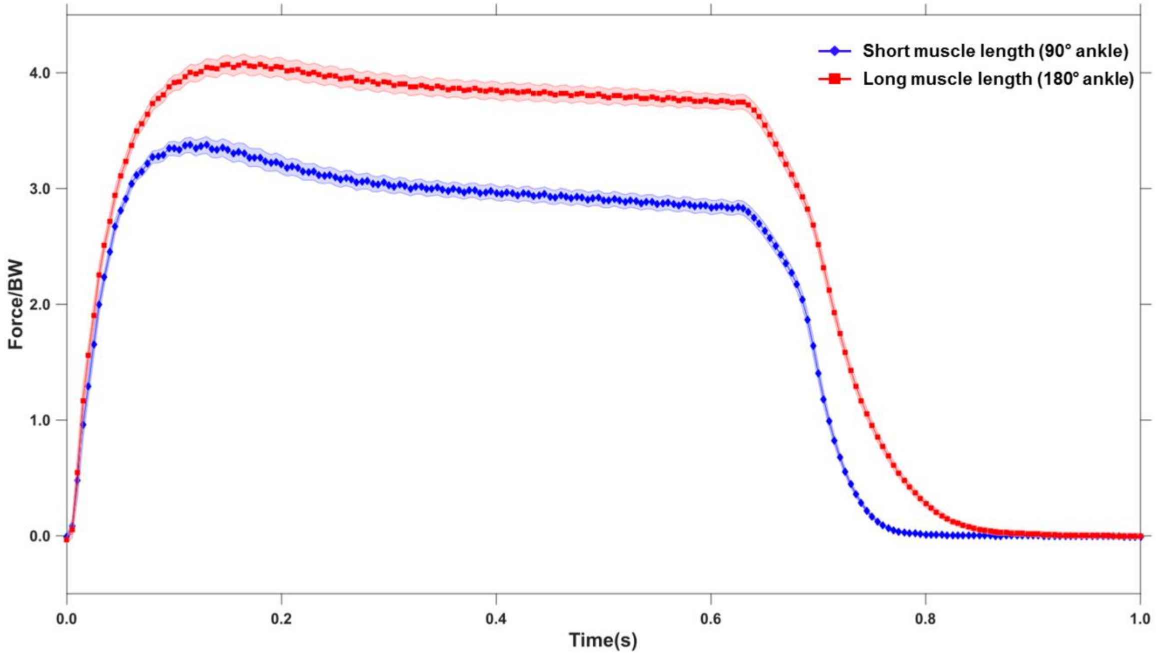

Fig. R17. Forces recorded at short and long muscle lengths for 'animal 7'.

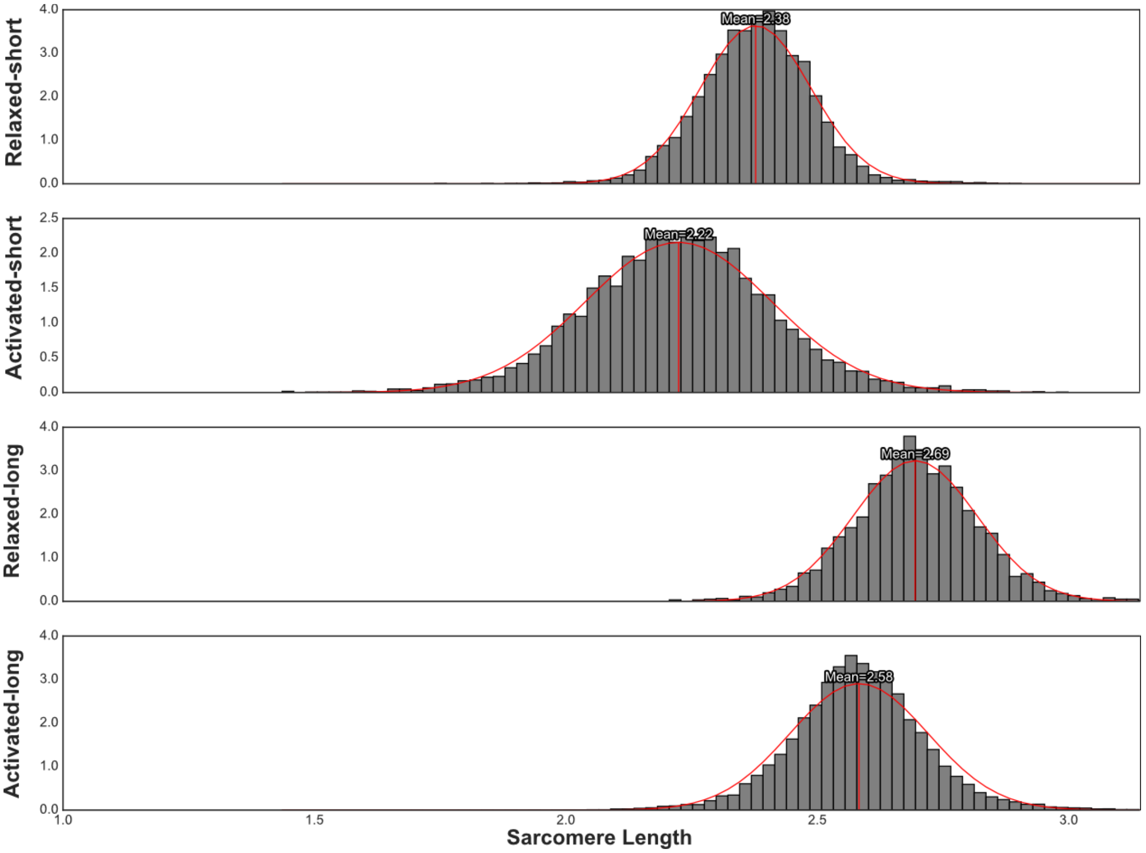

Fig. R18. SL PDF measured at short and long muscle lengths under relaxed and activated conditions for 'animal 7'.

Animal ID: 8

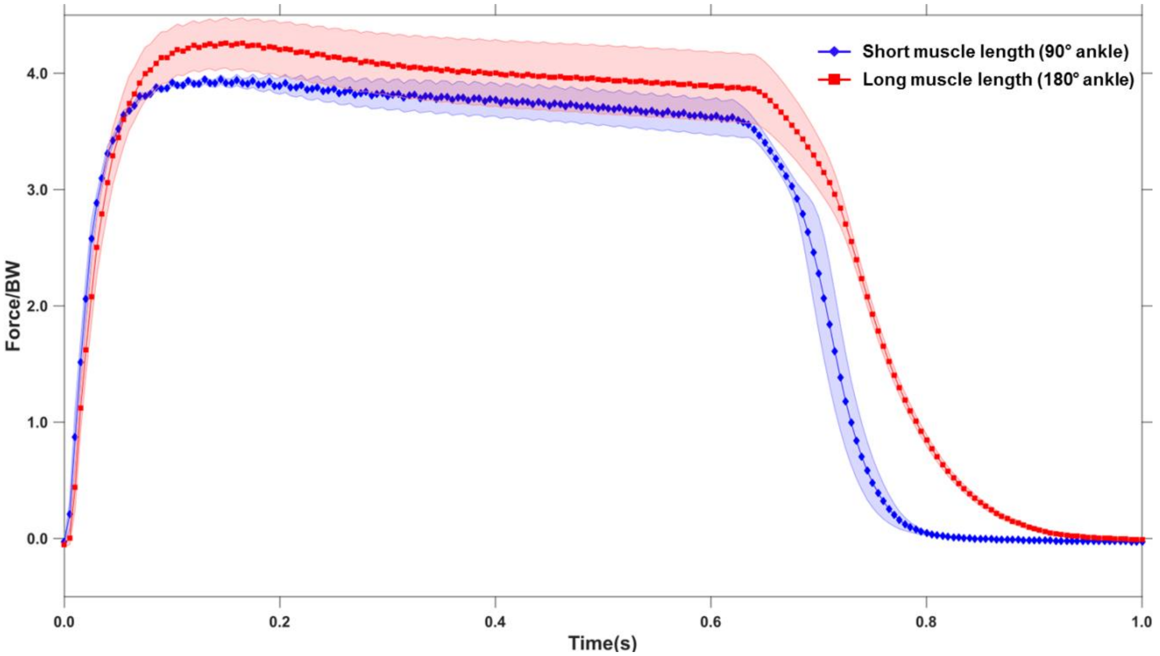

Fig. R19. Forces recorded at short and long muscle lengths for ‘animal 8’.

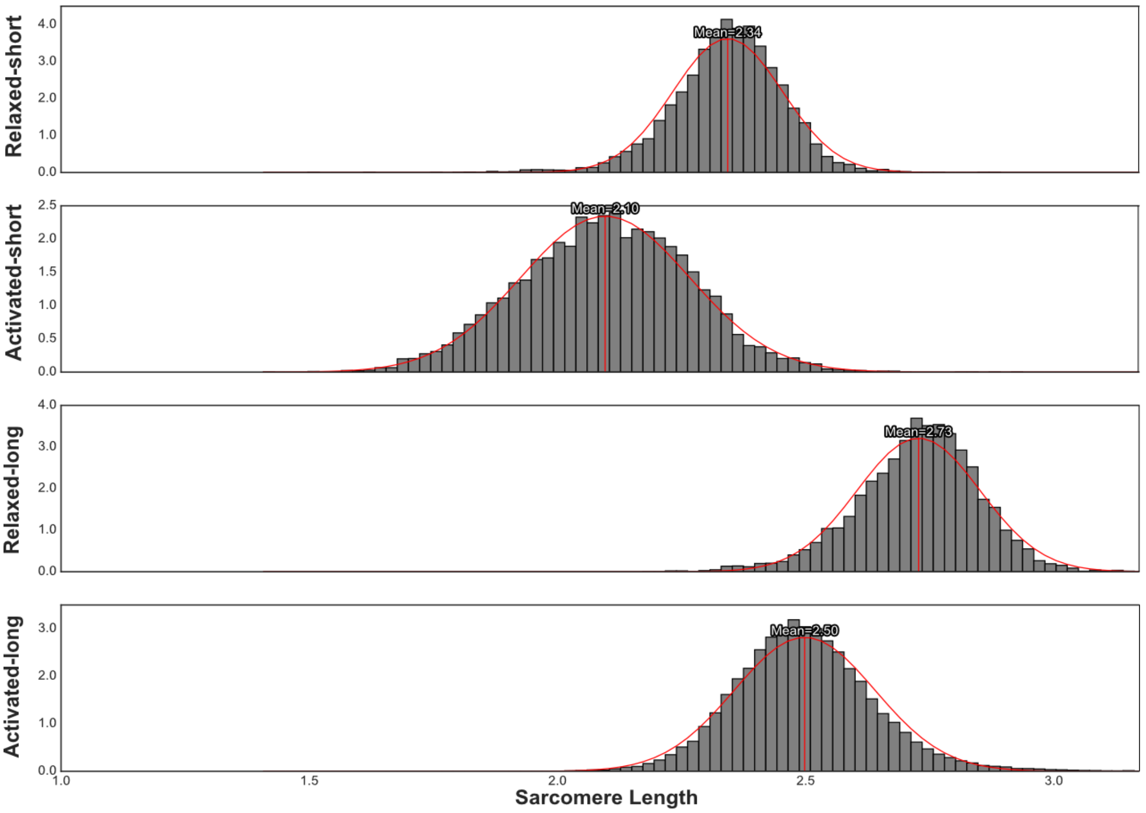

Fig. R20. SL PDF measured at short and long muscle lengths under relaxed and activated conditions for ‘animal 8’.

Animal ID: 9

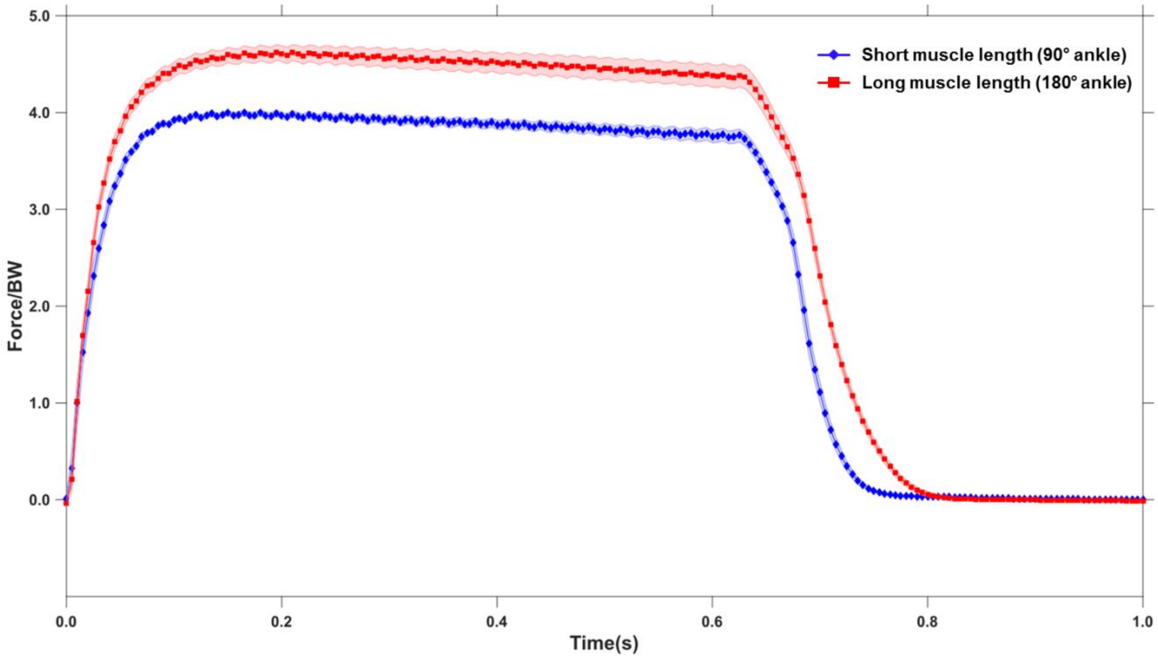

Fig. R21. Forces recorded at short and long muscle lengths for ‘animal 9’.

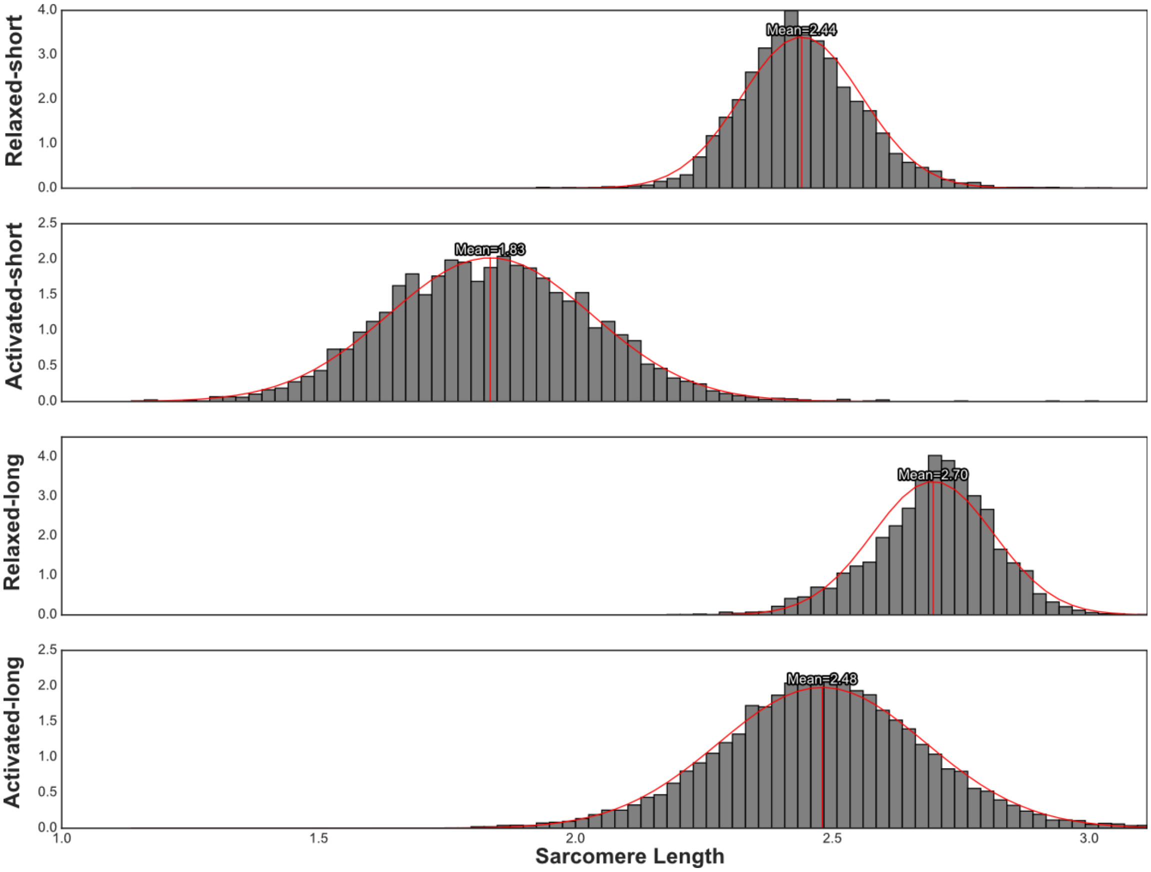

Fig. R22. SL PDF measured at short and long muscle lengths under relaxed and activated conditions for ‘animal 9’.
